# Neuroimaging-based data-driven subtypes of spatiotemporal atrophy due to Parkinson’s disease

**DOI:** 10.1093/braincomms/fcaf146

**Published:** 2025-04-16

**Authors:** Zeena Shawa, Cameron Shand, Beatrice Taylor, Henk W Berendse, Chris Vriend, Tim D van Balkom, Odile A van den Heuvel, Ysbrand D van der Werf, Jiun-jie Wang, Chih-Chien Tsai, Jason Druzgal, Benjamin T Newman, Tracy R Melzer, Toni L Pitcher, John C Dalrymple-Alford, Tim J Anderson, Gaëtan Garraux, Mario Rango, Petra Schwingenschuh, Melanie Suette, Laura M Parkes, Sarah Al-Bachari, Johannes Klein, Michele T M Hu, Corey T McMillan, Fabrizio Piras, Daniela Vecchio, Clelia Pellicano, Chengcheng Zhang, Kathleen L Poston, Elnaz Ghasemi, Fernando Cendes, Clarissa L Yasuda, Duygu Tosun, Philip Mosley, Paul M Thompson, Neda Jahanshad, Conor Owens-Walton, Emile d’Angremont, Eva M van Heese, Max A Laansma, Andre Altmann, ENIGMA Parkinson’s Disease Working Group, Max A Laansma, Joanna K Bright, Sarah Al-Bachari, Tim J Anderson, Tyler Ard, Francesca Assogna, Katherine A Baquero, Henk W Berendse, Benajmin Newman, Fernando Cendes, John C Dalrymple-Alford, Rob M A de Bie, Ines Debove, Michiel F Dirkx, Jason Druzgal, Hedley C A Emsley, Gäetan Garraux, Rachel P Guimarães, Boris A Gutman, Rick C Helmich, Johannes C Klein, Clare E Mackay, Corey T McMillan, Tracy R Melzer, Laura M Parkes, Fabrizio Piras, Toni L Pitcher, Kathleen L Poston, Mario Rango, Letícia F Ribeiro, Cristiane S Rocha, Christian Rummel, Lucas S R Santos, Reinhold Schmidt, Petra Schwingenschuh, Gianfranco Spalletta, Letizia Squarcina, Odile A van den Heuvel, Chris Vriend, Jiun-Jie Wang, Daniel Weintraub, Roland Wiest, Clarissa L Yasuda, Neda Jahanshad, Paul M Thompson, Ysbrand D van der Werf, Rimona S Weil, Neil P Oxtoby

**Affiliations:** UCL Hawkes Institute and Department of Medical Physics and Biomedical Engineering, University College London, London WC1E 6BT, United Kingdom; UCL Hawkes Institute and Department of Computer Science, University College London, London WC1E 6BT, United Kingdom; UCL Hawkes Institute and Department of Computer Science, University College London, London WC1E 6BT, United Kingdom; Department of Neurology, Amsterdam Neuroscience, Amsterdam UMC, Vrije Universiteit Amsterdam, Neurodegeneration, 1081 Amsterdam, The Netherlands; Department of Psychiatry, Amsterdam UMC, Vrije Universiteit Amsterdam, 1081 Amsterdam, The Netherlands; Department of Anatomy & Neurosciences, Amsterdam Neuroscience, Amsterdam UMC, Vrije Universiteit Amsterdam, Compulsivity Impulsivity & Attention, 1081 Amsterdam, The Netherlands; Department of Psychiatry, Amsterdam UMC, Vrije Universiteit Amsterdam, 1081 Amsterdam, The Netherlands; Department of Anatomy & Neurosciences, Amsterdam Neuroscience, Amsterdam UMC, Vrije Universiteit Amsterdam, Compulsivity Impulsivity & Attention, 1081 Amsterdam, The Netherlands; Department of Psychiatry, Amsterdam UMC, Vrije Universiteit Amsterdam, 1081 Amsterdam, The Netherlands; Department of Anatomy & Neurosciences, Amsterdam Neuroscience, Amsterdam UMC, Vrije Universiteit Amsterdam, Compulsivity Impulsivity & Attention, 1081 Amsterdam, The Netherlands; Department of Anatomy & Neurosciences, Amsterdam Neuroscience, Amsterdam UMC, Vrije Universiteit Amsterdam, Compulsivity Impulsivity & Attention, 1081 Amsterdam, The Netherlands; Department of Medical Imaging and Radiological Sciences, Chang Gung University, Taoyuan 33302, Taiwan; Department of Diagnostic Radiology, Chang Gung Memorial Hospital, Keelung 204, Taiwan; Healthy Aging Research Center, Chang Gung University, Taoyuan 33302, Taiwan; Department of Radiology and Medical Imaging, University of Virginia, Charlottesville, VA 22903, USA; Department of Radiology and Medical Imaging, University of Virginia, Charlottesville, VA 22903, USA; Department of Medicine, University of Otago, Christchurch 8011, New Zealand; New Zealand Brain Research Institute, Christchurch 8011, New Zealand; Te Kura Mahi ā-Hirikapo, School of Psychology, Speech and Hearing, University of Canterbury, Christchurch 8041, New Zealand; Department of Medicine, University of Otago, Christchurch 8011, New Zealand; New Zealand Brain Research Institute, Christchurch 8011, New Zealand; Department of Medicine, University of Otago, Christchurch 8011, New Zealand; New Zealand Brain Research Institute, Christchurch 8011, New Zealand; Te Kura Mahi ā-Hirikapo, School of Psychology, Speech and Hearing, University of Canterbury, Christchurch 8041, New Zealand; Department of Medicine, University of Otago, Christchurch 8011, New Zealand; New Zealand Brain Research Institute, Christchurch 8011, New Zealand; Department of Neurology, Christchurch Hospital, Te Whatu Ora Health NZ, Waitaha Canterbury 8140, New Zealand; MoVeRe Group, CRC Human Imaging, GIGA Interdisciplinary Biomedical Research Institute, University of Liege, 4000 Liege, Belgium; Neurology Unit, Excellence Interdepartmental Center for Advanced Magnetic Resonance Techniques, Fondazione Ca’ Granda, IRCCS, Policlinico, University of Studies of Milano, Milano 20122, Italy; Department of Neurology, Medical University of Graz, 8036 Graz, Austria; Department of Neurology, Medical University of Graz, 8036 Graz, Austria; Division of Psychology, Communication and Human Neuroscience, School of Health Sciences, Faculty of Biology, Medicine and Health, University of Manchester, Manchester M13 9PL, UK; Geoffrey Jefferson Brain Research Centre, Faculty of Biology, Medicine and Health, University of Manchester, Salford M6 8HD, UK; Department of Clinical and Movement Neurosciences, UCL, London WC1E 6BT, UK; Nuffield Department of Clinical Neurosciences (NDCN), University of Oxford, Oxford OX3 9DU, UK; Nuffield Department of Clinical Neurosciences (NDCN), University of Oxford, Oxford OX3 9DU, UK; Department of Neurology, University of Pennsylvania, Philadelphia, PA 19104, United States; Laboratory of Neuropsychiatry, Department of Clinical Neuroscience and Neurorehabilitation, Santa Lucia Foundation IRCCS, 00179 Rome, Italy; Laboratory of Neuropsychiatry, Department of Clinical Neuroscience and Neurorehabilitation, Santa Lucia Foundation IRCCS, 00179 Rome, Italy; Laboratory of Neuropsychiatry, Department of Clinical Neuroscience and Neurorehabilitation, Santa Lucia Foundation IRCCS, 00179 Rome, Italy; Ruijin Hospital, Shanghai Jiaotong University School of Medicine, Clinical Neuroscience Center, Shanghai 200031, China; Department of Neurology & Neurological Sciences, Stanford University, Stanford, Palo Alto, CA 94304, USA; Department of Neurology & Neurological Sciences, Stanford University, Stanford, Palo Alto, CA 94304, USA; Department of Neurology, University of Campinas—UNICAMP, Campinas 13083-872, Brazil; Brazilian Institute of Neuroscience and Neurotechnology, University of Campinas—UNICAMP, Campinas 13083-888, Brazil; Department of Neurology, University of Campinas—UNICAMP, Campinas 13083-872, Brazil; Brazilian Institute of Neuroscience and Neurotechnology, University of Campinas—UNICAMP, Campinas 13083-888, Brazil; Department of Radiology and Biomedical Imaging, University of California San Francisco, San Francisco, CA 94143, USA; QIMR Berghofer Medical Research Institute, Herston, QLD 4006, Australia; Imaging Genetics Center, Mark and Mary Stevens Institute for Neuroimaging & Informatics, Keck School of Medicine, University of Southern California, Los Angeles, CA 90033, USA; Laboratory of Brain eScience, Mark and Mary Stevens Neuroimaging and Informatics Institute, Keck School of Medicine of USC, Department of Biomedical Engineering, Viterbi School of Engineering, University of Southern California, Los Angeles, CA 90292, USA; Imaging Genetics Center, Mark and Mary Stevens Institute for Neuroimaging & Informatics, Keck School of Medicine, University of Southern California, Los Angeles, CA 90033, USA; Department of Anatomy & Neurosciences, Amsterdam Neuroscience, Amsterdam UMC, Vrije Universiteit Amsterdam, Neurodegeneration, 1081 Amsterdam, The Netherlands; Department of Anatomy & Neurosciences, Amsterdam Neuroscience, Amsterdam UMC, Vrije Universiteit Amsterdam, Neurodegeneration, 1081 Amsterdam, The Netherlands; Department of Anatomy & Neurosciences, Amsterdam Neuroscience, Amsterdam UMC, Vrije Universiteit Amsterdam, Neurodegeneration, 1081 Amsterdam, The Netherlands; UCL Hawkes Institute and Department of Medical Physics and Biomedical Engineering, University College London, London WC1E 6BT, United Kingdom; Dementia Research Centre, Department of Neurodegeneration, UCL Queen Square Institute of Neurology, University College London, London W1T 7NF, United Kingdom; UCL Hawkes Institute and Department of Computer Science, University College London, London WC1E 6BT, United Kingdom

**Keywords:** disease progression modelling, ENIGMA-PD, PPMI, neurodegeneration, clustering

## Abstract

Parkinson’s disease is the second most common neurodegenerative disease. Despite this, there are no robust biomarkers to predict progression, and understanding of disease mechanisms is limited. We used the Subtype and Stage Inference algorithm to characterize Parkinson’s disease heterogeneity in terms of spatiotemporal subtypes of macroscopic atrophy detectable on T1-weighted MRI—a successful approach used in other neurodegenerative diseases. We trained the model on covariate-adjusted cortical thicknesses and subcortical volumes from the largest known T1-weighted MRI dataset in Parkinson’s disease, Enhancing Neuroimaging through Meta-Analysis consortium Parkinson’s Disease dataset (*n* = 1100 cases). We tested the model by analyzing clinical progression over up to 9 years in openly-available data from people with Parkinson’s disease from the Parkinson’s Progression Markers Initiative (*n* = 584 cases). Under cross-validation, our analysis supported three spatiotemporal atrophy subtypes, named for the location of the earliest affected regions as: ‘*Subcortical*’ (*n* = 359, 33%), ‘*Limbic*’ (*n* = 237, 22%) and ‘*Cortical*’ (*n* = 187, 17%). A fourth subgroup having sub-threshold/no atrophy was named ‘*Sub-threshold atrophy*’ (*n* = 317, 29%). Statistical differences in clinical scores existed between the no-atrophy subgroup and the atrophy subtypes, but not among the atrophy subtypes. This suggests that the prime T1-weighted MRI delineator of clinical differences in Parkinson’s disease is atrophy severity, rather than atrophy location. Future work on unravelling the biological and clinical heterogeneity of Parkinson’s disease should leverage more sensitive neuroimaging modalities and multimodal data.

## Teaser

Shawa *et al.* used a large MRI dataset and machine-learning algorithm to identify three spatiotemporal atrophy subtypes of Parkinson's, the second most common neurodegenerative disease. Clinical phenotypes in a large external dataset differed from a pre-atrophy subgroup, but not from each other.

## Graphical Abstract

**Graphical Abstract:**
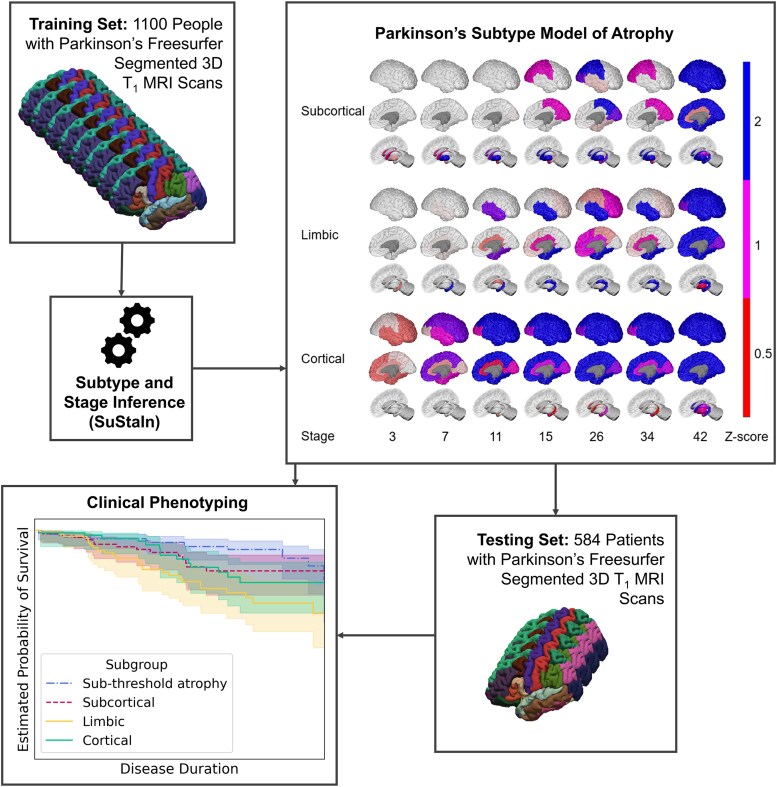

## Introduction

Parkinson’s disease is the second most prevalent neurodegenerative disease after Alzheimer’s disease, affecting >6 million people worldwide and growing rapidly,^1,2^ with the global burden (in terms of deaths and levels of disability) having doubled over the past two decades.^3^ Despite this, understanding of Parkinson’s disease progression remains limited, likely due to high levels of heterogeneity.^4^ The symptomatic treatments currently available are unable to halt, nor slow, the effects of disease progression.^5^ Most individuals will experience a reduced quality of life and eventual carer-dependency for day-to-day activities, especially due to neuropsychiatric symptoms and cognitive decline.^6,7^

Detection of misfolded alpha-synuclein is now feasible, but it lacks quantitation,^8^ which is needed to predict the risk of poor outcomes in and track the progression of Parkinson’s disease.^5^ This barrier to the development of disease-modifying therapies is likely due in part to disease heterogeneity and multiple underlying mechanisms. Indeed, a single biomarker that is dynamic throughout the decades-long duration of Parkinson’s disease progression may not exist. Progression may occur as a pathophysiological cascade of events, similar to the hypothetical model of Alzheimer’s disease.^9,10^ This model has seen subsequent support from data-driven models^11^ of both a predominant cascade of overall progression,^12,13^ and multiple cascades of different subtypes, i.e. data-driven subtypes of disease progression.^14,15^ There have been some data-driven explorations into Parkinson’s disease progression as a pathophysiological cascade,^16-19^ but little correspondence has been demonstrated between clinical phenotypes and imaging or fluid measurements in data-driven subtyping of the disease^20-23^. This may be explained by relying on relatively small datasets,^21^ having limited clinical or neuroimaging information,^20^ or lacking longitudinal progression information^20-22^ beyond motor progression.^23^

There is great interest in redefining Parkinson’s disease into progression subtypes, including examining non-motor symptom progression, as this may reveal quantitative biomarkers to inform the design of clinical trials^24-28^ for disease-modifying therapies. Indeed, the National Institute of Neurological Disorders and Stroke has listed subtype identification as one of the top three clinical research priorities in Parkinson’s disease.^29^ Subtyping approaches to date have predominantly relied on empirical clinical phenotyping.^25,30^ These categorize people with Parkinson’s using motor symptoms (e.g. tremor-dominant or non-tremor-dominant),^31^ presence of mild cognitive impairment,^30,32,33^ age of onset (young/old)^34^ and rate of disease progression (fast/slow decline).^35^ The increasing availability of large multimodal datasets presents an opportunity to go beyond empirical approaches and deploy advanced neuroimaging analyses and the latest developments in medical data science such as disease progression modelling.^11^

Unsupervised machine learning methods, such as clustering,^24,25^ have been used to identify subtypes in Parkinson’s disease—mostly driven by clinical symptoms as input features. Using these or similar approaches, several groups have identified subtypes with distinct progression rates from the Parkinson’s Progression Markers Initiative (PPMI).^24,36-40^ Dadu *et al.*^37^ (*n* = 294, validated in *n* = 263) used a combination of supervised clustering methods to find three distinct clinical progression subtypes—slow, moderate and fast. Erro *et al.*^36^ also found three clusters in the PPMI dataset (*n* = 398), using non-hierarchical cluster analysis, which were characterized by differing motor and non-motor burden. Fereshtehnejad *et al.*^24^ similarly found three subtypes in the PPMI data set (*n* = 421), using hierarchical cluster analysis. These were named mild-motor predominant, diffuse malignant and intermediate. The diffuse malignant subtype had increased motor and cognitive decline, greater dopaminergic deficit, increased atrophy in Parkinson’s disease brain networks and a CSF profile resembling Alzheimer’s disease. Su *et al.*^38^ used unsupervised deep learning, followed by hierarchical clustering in a latent space, revealing three subtypes distinguished by the speed of symptom progression (mild, moderate, rapid). They reported associations with imaging, CSF, genetic and transcriptomic data in these clinically-driven subtypes. Zhang *et al.*^39^ (*n* = 466) used a deep learning algorithm (Long-Short Term Memory network) to create a multi-dimensional time series representation of each person with Parkinson’s to inform the subtyping. They also found three subtypes characterized by different baseline states, and differing clinical progression. More recently, Severson *et al.*^40^ (*n* = 423) found eight non-sequential, overlapping disease states using a latent variable hidden Markov model. Between the states, non-motor symptoms fluctuate in severity, while motor symptoms show a more consistent increase in severity with overall symptom progression.

Distinct subtypes have also been found using similar approaches in other cohorts. For example, model-based cluster analysis conducted by van Rooden *et al.*^35^ in a Dutch Parkinson’s disease cohort (*n* = 344) discovered four subtypes: mildly affected, severe motor, nondopaminergic domains affected and severely affected. These subtypes had clinical and demographic differences, and results were reproduced in a second dataset (*n* = 357). Lawton *et al.*^41^ used factor analysis and k-means clustering to identify subtypes by differences in motor, cognitive and non-motor domains. They found four subtypes, with moderate replication across two cohorts (*n* = 1601 and *n* = 944), with differing rates of motor progression and levodopa treatment response.

Despite substantial research on subtyping and disease progression, few studies validate their findings with external datasets, incorporate MRI data in their models and demonstrate reproducible clinical applicability or biological relevance.^19,26,40,42,43^ Moreover, most previous studies including imaging compare imaging associations across pre-defined clinical-based subtypes, rather than leveraging the available imaging data itself to identify clinical subtypes.^30-35,38,40,44-46^ Here, we aim to add to the Parkinson’s subtyping literature by discovering neuroimage-based subtypes of Parkinson’s disease progression using the largest known T1-weighted MRI Parkinson’s dataset. We used the Subtype and Stage Inference (SuStaIn) algorithm,^14,47^ applied to imaging features extracted from T1-weighted MRI in the Enhancing Neuroimaging through Meta-Analysis consortium Parkinson’s Disease (ENIGMA-PD) dataset,^48^ and validated in the PPMI dataset. SuStaIn combines data-driven disease progression modelling^11^ with clustering to unravel spatial and temporal (progression) heterogeneity into disease progression subtypes. SuStaIn has previously determined clinically-useful image-based subtypes in other neurodegenerative diseases, such as Alzheimer’s disease,^14,15,49,50^ multiple sclerosis^51^ and frontotemporal dementia.^52^ This is a data-driven progression-subtyping study in a uniquely large collection of people with Parkinson’s disease.

## Materials and methods

**Figure 1:**
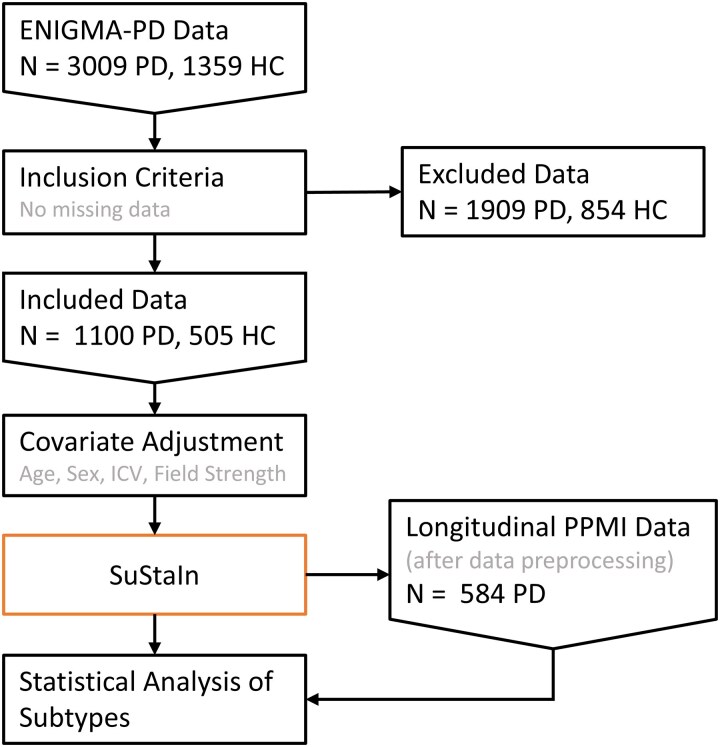
**Study flowchart.** The ENIGMA-PD data used for training was selected based on having no missing data in age, sex, disease duration and imaging features. Excluded individuals had a median of 6 missing features of interest, with a MAD of 2. Cortical thickness measurements were the most frequently absent feature by a large margin (Supplementary Fig. 8). Covariate adjusted imaging features were the inputs to the SuStaIn algorithm. The trained model was used to subtype and stage PPMI participants, in whom demographic and longitudinal clinical outcomes were compared. SuStaIn, Subtype and Stage Inference; HC, healthy control; ICV, intracranial volume; PD, Parkinson’s disease; ENIGMA, Enhancing Neuroimaging through Meta-Analysis; PPMI, Parkinson’s Progression Markers Initiative; MAD, median absolute deviation.

Figure 1 shows a schematic of this study. Briefly, we collected brain volume and cortical thickness features from two large datasets of people with Parkinson’s disease, *z*-score normalized these features with respect to a large dataset of healthy controls and adjusted them for confounders, namely age, sex, intracranial volume (ICV) and MRI scanner field strength. These features of the ENIGMA-PD dataset were then used as inputs to the SuStaIn algorithm to discover group-level atrophy subtypes of Parkinson’s disease. The second external test dataset, PPMI,^53^ was used to explore clinical progression across the image-based subtypes.

### Data selection and preparation

#### Participants

The model training set includes data from the multi-site international ENIGMA-PD working group.^48^ The external test dataset used to analyse longitudinal progression of data-driven subtypes consists of openly-available multi-site data from PPMI, downloaded in January 2024.^54^

Training data: ENIGMA-PD contains cross-sectional neuroimaging markers and clinical data on people with Parkinson’s disease (diagnosed mostly using either Movement Disorders Society, National Institute of Neurological Disorders and Stroke, or UK Parkinson’s Disease Society Brain Bank criteria) and healthy controls collated between September 2016 and December 2019 from 23 sources, with unique inclusion and exclusion criteria described in the Supplementary material of Laansma *et al*.^48^ Some sources included multiple cohorts and/or centres, such as PPMI.^53^ In total, the cohort includes 1359 healthy controls and 3009 people with Parkinson’s disease. Inclusion criteria for our study was having complete demographic and imaging data: age, sex, disease duration, select regional cortical thickness and subcortical volume values (described below) and ICV. Data from individuals with outliers in ICV (beyond the median ± 3*interquartile range) were excluded. All PPMI individuals were removed from the training set, to prevent any data leakage between training and test sets. The training set included data from 1100 people with Parkinson’s disease (63.1 ± 9.3 years old; 37.6% female) and 505 healthy controls (61.1 ± 10.5 years old; 48.9% female).

Test data: PPMI is a longitudinal and international multi-centre data set with clinical, genetic, neuroimaging and blood/CSF biomarkers from over 900 people with Parkinson’s disease diagnosed within 2 years of enrolment and previously unmedicated. The details of the PPMI study setting and population can be found in works by Marek *et al.*^53,55^ People with Parkinson’s disease were clinically assessed both off and on their dopaminergic therapy, except during cognitive assessments (‘on state’).^56^ We analysed only ‘off state’ non-cognitive data to avoid confounding by symptomatic treatments. After excluding outliers in ICV (beyond median ±3×interquartile range) and applying our study inclusion criteria, the test set included 584 people with Parkinson’s disease (39.4% female) aged 63.1 ± 9.5 years old. Eighty-seven of these individuals had genetic variants linked with Parkinson’s disease. Table 1 summarizes the demographics for both datasets.

**Table 1:** Baseline descriptive statistics of the data

	Training: ENIGMA-PD		Test: PPMI	*P*-value^a^
	Healthy Controls	Parkinson’s disease	Parkinson’s disease	
Number, *n*	505	1100	584	**<0**.**0001**
% Female	48.9	37.6	39.4	0.52
Age, years ± SD	61.1 ± 10.5	63.1 ± 9.3	63.1 ± 9.5	0.45
Age of Onset, years ± SD (*n*)	–	56.5 ± 10.9 (717)	62.0 ± 9.6 (571)	1.00
Disease duration, years ± SD (*n*)	–	7.4 ± 5.5 (871)	0.9 ± 1.2 (571)	**<0**.**0001**
Mean follow-up duration, years ± SD (*n*)	–	–	4.3 ± 3.6 (584)	–
Education, years ± SD (*n*)	16.1 ± 2.4 (18)	14.4 ± 3.8 (36)	14.1 ± 3.1 (12)	0.31
Hoehn and Yahr Stage, median [MAD] (*n*)	–	2.0 [0.0] (730)	2.0 [0.0] (580)	**<0**.**0001**
MoCA, mean ± SD (*n*)	27.9 ± 1.6 (138)	23.6 ± 4.9 (588)	26.9 ± 2.6 (573)	1.00
Total Motor Score (MDS-UPDRS-III), mean ± SD (*n*)	1.2 ± 1.9 (8)	31.6 ± 14.7 (118)	22.2 ± 10.0 (579)	**<0**.**0001**

^a^ *P*-values from statistical tests comparing the Parkinson’s disease groups from each dataset. Bolded text indicates a *P*-value ≤ 0.05. Mann–Whitney *U* test for all, apart from ‘Number’, % Female, and ‘Hoehn and Yahr Stage’, which were obtained with a Pearson’s *χ*^2^.

ENIGMA-PD, Enhancing Neuroimaging through Meta-Analysis consortium Parkinson’s Disease; MDS-UPDRS, Movement Disorders Society Unified Parkinson’s disease Rating Scale; MoCA, Montreal Cognitive Assessment; PPMI, Parkinson’s Progression Markers Initiative; SD, standard deviation; MAD, median absolute deviation.

Parkinson’s disease diagnosis in ENIGMA-PD followed local protocols and individual site inclusion and exclusion criteria are detailed in Laansma *et al*.^48^ All people with Parkinson’s disease in PPMI matched the following inclusion criteria: (i) asymmetric resting tremor or asymmetric bradykinesia, or two of bradykinesia, resting tremor and rigidity; (ii) a clinical diagnosis of PD within 2 years of recruitment; (iii) untreated; (iv) did not have dementia; and (v) had a positive dopamine transporter SPECT.^57^ Recruited healthy controls had no first-degree family with PD, no neurological dysfunction and a Montreal Cognitive Assessment (MoCA) score greater than 26. All PPMI participants provided informed consent, including for secondary analyses such as ours. Detailed inclusion and exclusion criteria for PPMI are outlined in documentation by Marek *et al*.^58^ All data used were deidentified. Full ethical approval was obtained from every site’s respective local ethics committee and institutional review board. Our secondary analysis was approved by the UCL Research Ethics Committee (8019/005).

#### MRI acquisition and data pre-processing

All participants underwent a 3-dimensional gradient-echo T1-weighted structural brain MRI scan. Site-specific parameters are detailed in Laansma *et al.*^48^ and the PPMI manual.^56^ FreeSurfer version 5.3 was used to extract ICV, cortical thickness, cortical surface area and subcortical volume measures from the structural MRI scans,^59,60^ using the Desikan−Killiany atlas.^61,62^ As in previous work,^14^ the 34 cortical regions of interest per hemisphere were grouped into five lobes^63^ plus the insula. This ensured a sufficiently low number of input features to make model training time achievable—for SuStaIn, this increases exponentially with added features. These six cortical thicknesses and the volumes of eight subcortical regions of interest (per hemisphere) were averaged across hemispheres to produce 14 bilateral imaging-derived phenotypes: frontal lobe, temporal lobe, parietal lobe, occipital lobe, cingulate and insula cortical thickness; and volumes of the putamen, caudate, thalamus, hippocampus, amygdala, pallidum, accumbens and lateral ventricles. The laterality or symmetry of neurodegeneration in Parkinson’s disease is debated in the literature.^64^ Our study found that group-level atrophy patterns were symmetric—initial experiments with separate left- and right-brain imaging biomarkers (not shown) discovered symmetrical atrophy subtypes. Thus, we combined left and right into a single measure per region, which had computational and interpretational benefits.

Regression-based covariate adjustment was used to remove confounding non-disease related effects of ICV, MRI field strength, age and biological sex on the imaging features. First, a General Linear Model was used to learn these confounding trends in training data from healthy controls, using the following formula:


(1) y∼Age+Sex+ICV+FieldStrength

where y is a given imaging feature. The healthy trends were then subtracted from the raw data before being converted to input features for SuStaIn. This process did not include study site (such as in neuroCombat harmonisation)^65^ due to insufficient data at many sites.^65^

### Disease progression modelling and subtyping

The open-source Python implementation of the SuStaIn algorithm^14,47^ (pySuStaIn v0.1, Python 3.8.1) was used to train a computational model of Parkinson’s disease atrophy subtypes. SuStaIn is a probabilistic unsupervised machine learning technique^14^ that combines clustering and disease progression modelling to discover subtypes having distinct biomarker progression patterns. The progression patterns here correspond to cumulative atrophy, quantified as covariate-adjusted *z*-scores. SuStaIn infers *progression subtypes*, from which it can assign the most likely (best matching) subtype and stage to participant data.^14,47^ Stages correspond to the probabilistic order in which biomarkers become cumulatively abnormal at selected levels of severity (see below).

The model was trained on cross-sectional Parkinson’s disease participant data from ENIGMA-PD, with subtypes/clinical-phenotype relationships characterized using longitudinal participant data from PPMI (see Table 1). Cross-validation metrics, namely the Cross-Validation Information Criterion and out-of-sample log-likelihood, were used to inform the number of subtypes by comparing models having between *N_S_* = 1 up to *N_S_* = 9 subtypes.^14,66^ Markov Chain Monte Carlo sampling from the posterior (10 000 iterations) characterized uncertainty in subtype atrophy, as described in Aksman *et al.*^47^ Participant imaging derived phenotype data were normalized into covariate-adjusted, robust *z*-scores using the median and median absolute deviation in data from controls. We set model hyperparameter waypoints to be cumulative *z*-score atrophy events of *z* = [0.5,1,2], corresponding to 69.1, 84.1 and 97.7% of a normal cumulative density function, respectively. These choices were motivated by early detection—a *z*-score of 0.5 representing subtle atrophy. The progression subtypes were visualized with positional variance diagrams^67^ and BrainPainter.^68^

#### Statistical analysis

The trained subtype model was deployed to assign subtype and stage to baseline data in the test set. The model produces a probability for each subtype and stage, where the final subtype and stage an individual is assigned is based on the subtype and stage with maximum probability. Subtypes were compared statistically using appropriate statistical tests (described below), with outcomes including demographics and longitudinal clinical assessments: cognitive score (MoCA), autonomic function (Scales for Outcomes in Parkinson’s Disease Autonomic Dysfunction), motor score (Movement Disorders Society Unified Parkinson’s Disease Rating Scale: Part III Motor Examination, MDS-UPDRS-III), and REM-sleep behaviour disorder (Rapid Eye Movement Sleep Behaviour Disorder Screening Questionnaire). Longitudinal secondary clinical and psychological test scores included: MoCA delayed recall, attention, naming, orientation and visual sub-scores; Benton Judgment of Line Orientation; Symbol Digit Modality; Lexical Fluency; Semantic Fluency; Boston Naming; Letter Number Sequencing; Hopkins Verbal Learning Test; State-Trait Anxiety Inventory; and Geriatric Depression Scale. As well as at the baseline PPMI visit, cross-sectional comparisons were performed for Visit 4 (Year 1), Visit 6 (Year 2), Visit 8 (Year 3), Visit 10 (Year 4) and Visit 12 (Year 5).

Cross-sectional comparisons used the Kruskal–Wallis test (from the *pingouin* Python package^69^) followed by the Conover test for continuous variables (from the *scikit-posthocs* package^70^ and chosen over Dunn’s test as it is more powerful and appropriate for smaller sample sizes).^71-73^ Pearson’s *χ*^2^ test was used to compare the number of individuals in each subgroup.^74^ One-vs-all comparisons were performed using a Mann−Whitney *U* test.^74^

Longitudinal comparisons used linear mixed regression models (using the *statsmodels* package^75^) and survival analysis (using the *scikit-survival* package^76^). Since MoCA scores may be positively skewed due to participants dropping out, we performed a survival analysis corresponding to the earliest occurrence of either MoCA below 21 or any of the following reasons for withdrawal: burden of study procedures (other than travel), decline in health, institutionalized, death. Multiple (pairwise) subtype comparisons were Bonferroni-corrected where appropriate. MoCA below 21 is a widely used cutoff for detecting Parkinson’s disease dementia.^77,78^ Cox proportional hazards models, including age and sex as covariates, were used to determine whether subtypes had statistically different survival distributions from each other. Age of onset was adjusted for as a continuous value and as a stratified binary value based on a cutoff age of onset under 65 years. This was done to ensure that all model variables pass the non-proportional test and that the model meets the proportional hazards assumption. As in previous work,^17^ the cutoff was decided based on an approximate average Parkinson’s disease age of onset, which is estimated to be in the range of 60 to 70 years old.^79^ Longitudinal consistency of the subtypes model was assessed by the fraction of participant data being assigned the same subtype at baseline and at follow-up visits.

## Results

### Disease progression subtype model

**Figure 2:**
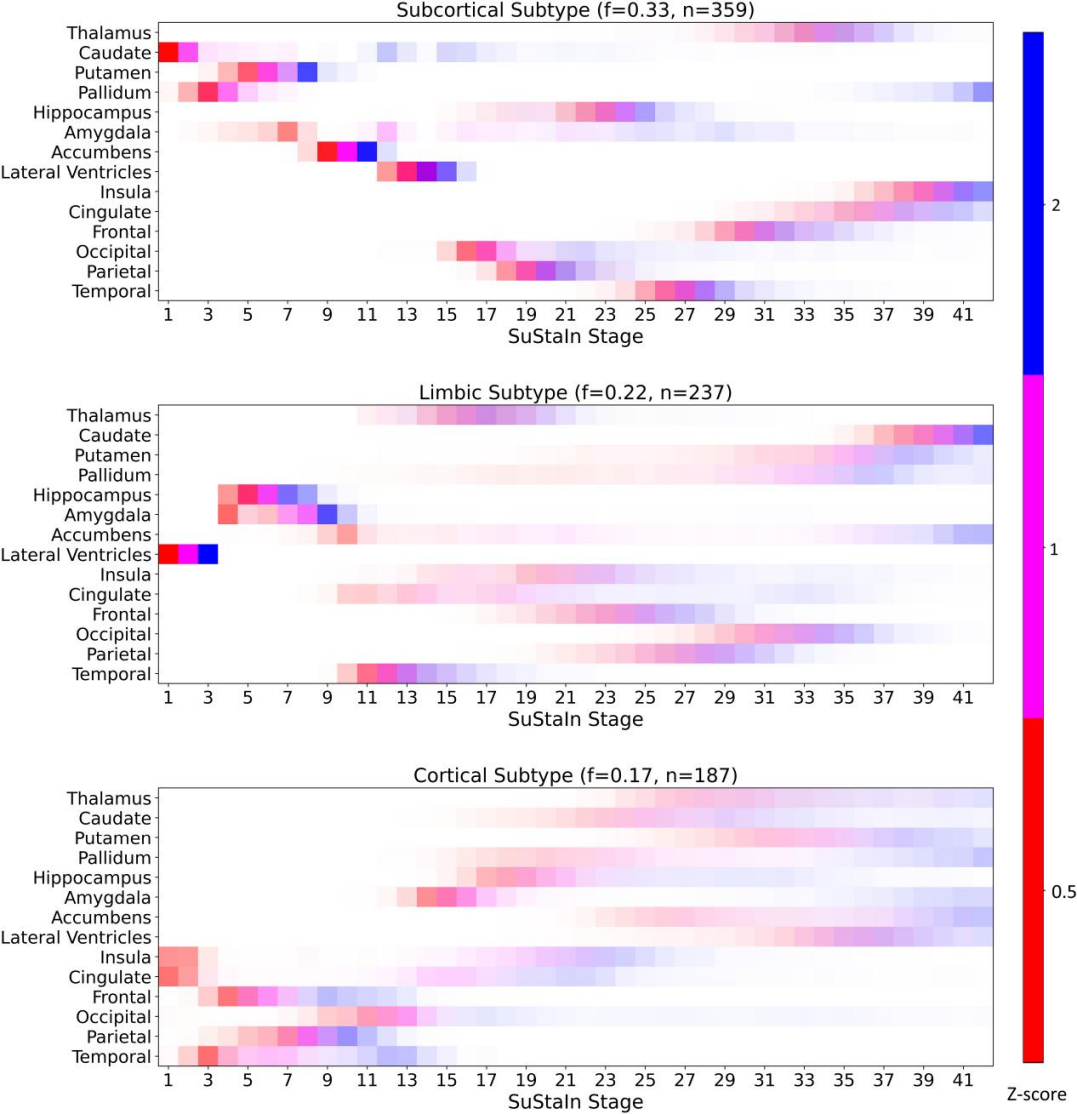
**Data-driven model of Parkinson’s disease atrophy subtypes.** Positional Variance Diagrams show accumulating atrophy (left-to-right) in brain regions (vertical axis), as estimated by SuStaIn on data from ENIGMA-PD. The SuStaIn stages correspond to the probabilistic order in which brain regions become abnormal, in disease pseudo time, compared with healthy controls. The model has 42 stages of cumulative abnormality corresponding to 3 *z*-score events per 14 input features. Subtypes are named *Subcortical*, *Limbic*, and *Cortical* for the location of the earliest atrophy events, which are colour-coded in red (mild, *z* = 0.5), magenta (moderate, *z* = 1) and blue (severe, *z* = 2). Colour intensity corresponds to the model confidence in atrophy event positions (stages) within each subtype sequence, where confidence is sharp, and uncertainty blurry. The fraction of patient data in the training set assigned to each subtype is indicated by f, with the remainder designated as the *Sub-threshold atrophy* subgroup. SuStaIn, Subtype and Stage Inference; PD, Parkinson’s disease; ENIGMA, Enhancing Neuroimaging through Meta-Analysis.

**Figure 3:**
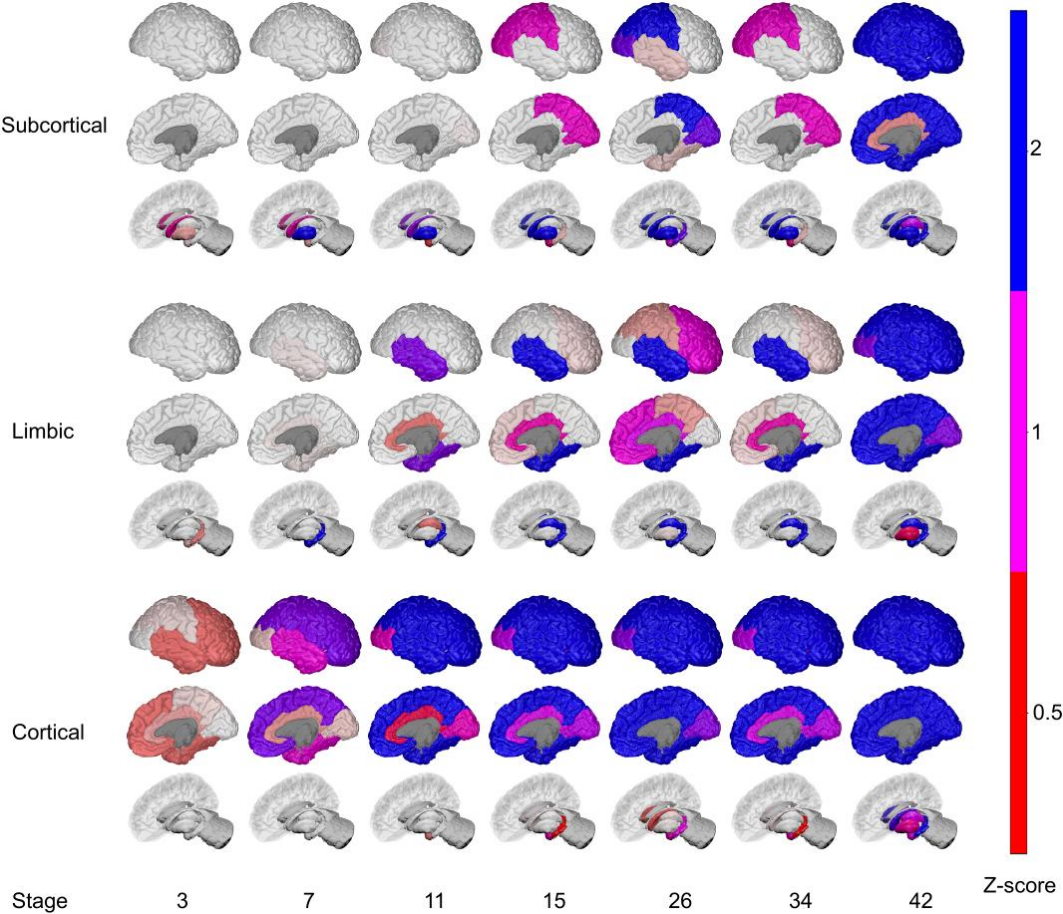
**Visualization of Parkinson’s disease atrophy subtypes model.** BrainPainter^68^ visualizations of the pattern of neurodegeneration. Colours indicate atrophy severity as *z*-scores relative to healthy controls: red (*z* = 0.5, mild), magenta (*z* = 1, moderate), blue (*z* = 2, severe). For simplicity, snapshots of only a few select stages (*x*-axis) are shown per subtype. Stages correspond to the probabilistic order in which brain regions become abnormal in disease pseudo time. Colour intensity corresponds to the model confidence in atrophy event positions (stages) within each subtype sequence, where confidence is sharp, and uncertainty blurry. Colours are blended linearly, indicating overlap between *z*-score events that overlap. An example of this can be seen at stage 26 of the *Subcortical* subtype, where magenta (z > 1) and blue (*z* > 2) mix.

Figure 2 shows the trained three-subtype model (determined using cross-validation, results in Supplementary Figs 1 and 2) as colour-coded positional variance diagrams,^67^ representing cumulative (left-to-right) atrophy events in red (mild atrophy, *z* = 0.5), magenta (moderate atrophy, *z* = 1) and blue (high atrophy, *z* = 2). Colour intensity corresponds to posterior probability density, i.e. model confidence (sharp) or uncertainty (blurry) in atrophy event positions within each subtype sequence. Features are ordered on the *y*-axis from subcortical (top) to cortical. Each subtype is named after distinctive early atrophy events: *Subcortical* (caudate, pallidum, putamen); *Limbic* (lateral ventricles, hippocampus, amygdala); *Cortical* (lobes). Figure 3 shows a visual representation of the subtype progression patterns on a model brain.

**Figure 4:**
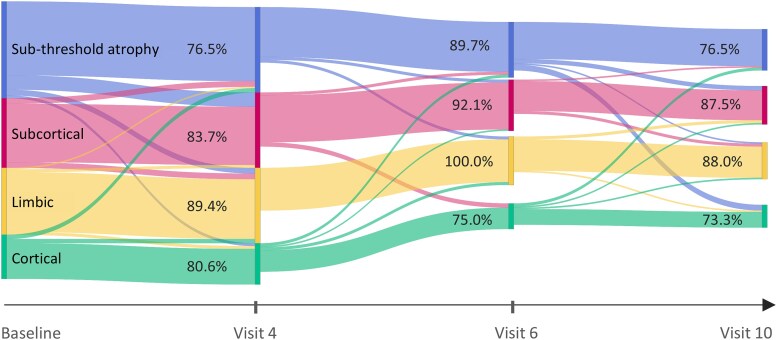
**Longitudinal consistency of subtypes in the PPMI test data.** A Sankey diagram showing PPMI subtype assignments by study visit. The percentages reflect individuals that are assigned the same subtype since Baseline. Most participant data are consistently assigned to a single subtype. PPMI, Parkinson’s Progression Markers Initiative.

Figure 4 is a Sankey Diagram visualizing the longitudinal consistency of subtype assignment for PPMI test data at each of four visits (where MRI data were available). The minimal crossover and comparable proportions at each study visit demonstrate high longitudinal consistency of subtype assignment—an average consistency of 84.4% (standard deviation 7.8%) across all visits, accounting for attrition—providing additional confidence that the Parkinson’s disease atrophy subtypes discovered by SuStaIn from cross-sectional data are longitudinally valid.

**Figure 5:**
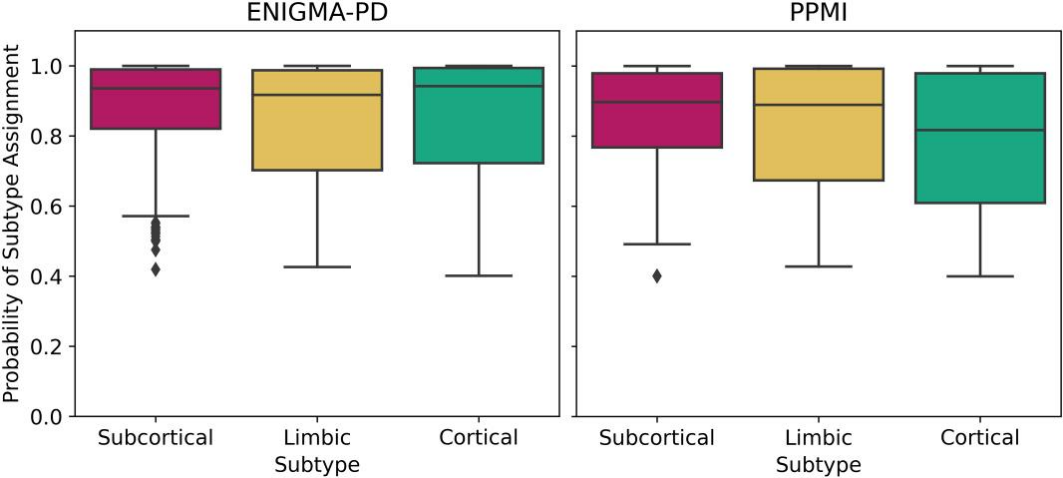
**Model-based subtype confidence.** Box plots show high confidence in subtype assignment (subtype probability) for most participants in both the training dataset (left: ENIGMA-PD, *N* = 359, 237 and 187 for subcortical, limbic and cortical, respectively) and the test dataset (right: PPMI, *N* = 152, 143 and 97 for subcortical, limbic and cortical respectively). PD, Parkinson’s disease; ENIGMA, Enhancing Neuroimaging through Meta-Analysis; PPMI, Parkinson’s Progression Markers Initiative.

Figure 5 shows confidence in subtype assignment for both the training set (left) and test set (right) via distributions of subtype probability. The 25th percentile for each distribution was between 0.65 and 0.85, indicating that subtype assignment is confident for most participants. The heat map of staging density per subtype in Supplementary Fig. 3 shows a skew towards early stages, often attributed to attrition.

### Cross-sectional subgroup analysis: ENIGMA-PD and PPMI

Table 2 compares subtype demographics and clinical features. The *Subcortical* atrophy group was the largest subtype, with the following subtype distribution sample sizes in the ENIGMA-PD training data: *Subcortical* (*n* = 359, 32.6%), *Limbic* (*n* = 237, 21.7%), *Cortical* (*n* = 187, 17%). The remaining 28.8% (*n* = 317) of ENIGMA-PD participants were classified into a sub-threshold atrophy subgroup having *z*  *<* 0.5 in all brain regions. This subgroup is referred to as ‘*Sub-threshold atrophy*’ due to individuals having minimal atrophy in all brain regions, comparable to controls. Subgroup proportions were statistically different between PPMI and ENIGMA-PD (*Sub-threshold atrophy* was proportionally larger and the *Subcortical* smaller in PPMI, Pearson’s *χ*^2^  *P* = 0.03). Statistically significant differences in both the training set and test set were observed in age (*Limbic* older, Kruskal–Wallis *P* < 0.001), age at onset (*Limbic* older, *Subcortical* younger, Kruskal–Wallis *P* < 0.01) and disease duration (*Sub-threshold atrophy* had a shorter disease duration, Kruskal–Wallis *P* < 0.01). Biological sex was also observed to be significantly different in the test set (*Cortical* had fewer females than expected, Pearson’s *χ*^2^, *P* = 0.02).

**Table 2:** Clinical features per subtype at baseline

Characteristic	Dataset	*Sub-threshold atrophy*	*Subcortical*	*Limbic*	*Cortical*	*P*-value^a^
Count	ENIGMA-PD	317	359	237	187	**0**.**03**
	PPMI	192	152	143	97	
% Female	ENIGMA-PD	38.8	40.9	33.3	34.8	0.22
	PPMI	34.9	37.5	34.3	19.6	**0.02**
Age, mean ± SD (count)	ENIGMA-PD	62.2 ± 8.6 (317)	61.9 ± 9.8 (359)	**66.3** ± **9.1 (237)**	63.0 ± 8.7 (187)	***<*0.001**
	PPMI	60.2 ± 9.8 (192)	61.4 ± 9.0 (152)	**67.7** **±** **8.9 (143)**	**64.5** **±** **7.8 (97)**	***<*0.001**
Age of Onset, mean ± SD (count)	ENIGMA-PD	57.0 ± 9.9 (207)	54.6 ± 11.1 (198)	**58.8** **±** **11.7 (163)**	55.8 ± 10.7 (149)	***<*0.01**
	PPMI	59.3 ± 9.9 (189)	60.2 ± 9.1 (149)	**66.7** **±** **9.0 (136)**	**63.5** **±** **7.7 (97)**	***<*0.001**
Disease Duration, Years ± SD (count)	ENIGMA-PD	**6.4** **±** **4.9 (249)**	8.0 ± 5.6 (284)	8.0 ± 5.9 (181)	7.2 ± 5.5 (157)	**<0.01**
	PPMI	0.8 ± 1.1 (189)	1.1 ± 1.5 (149)	0.9 ± 0.9 (136)	1.0 ± 1.0 (97)	**0.04**
Years of Education, mean ± SD (count)	ENIGMA-PD	15.9 ± 2.9 (75)	15.0 ± 3.5 (58)	15.6 ± 3.4 (46)	15.3 ± 3.3 (65)	0.48
	PPMI	− (3)	− (3)	− (6)		
Hoehn and Yahr Stage, median [MAD] (count)	ENIGMA-PD	2.0 [0.0] (199)	2.0 [0.0] (240)	2.0 [0.0] (143)	2.0 [0.0] (148)	***<*0.01**
	PPMI	2.0 [0.0] (190)	2.0 [0.0] (150)	2.0 [0.0] (143)	2.0 [0.0] (97)	0.12
MoCA Score, mean ± SD (count)	ENIGMA-PD	24.3 ± 4.4 (177)	23.8 ± 4.9 (214)	**22.2** **±** **5.3 (124)**	23.4 ± 5.1 (73)	**<0.01**
	PPMI	27.2 ± 2.5 (190)	26.9 ± 2.6 (147)	26.5 ± 2.9 (141)	27.0 ± 2.2 (95)	0.18
MDS-UPDRS-III, mean ± SD (count)	ENIGMA-PD	29.9 ± 13.8 (43)	33.0 ± 15.5 (48)	33.0 ± 12.8 (17)	29.6 ± 18.4 (10)	0.63
	PPMI	20.9 ± 9.3 (189)	23.0 ± 10.0 (150)	22.7 ± 10.3 (143)	22.7 ± 10.5 (97)	0.19
RBD Score, mean ± SD (count)	ENIGMA-PD					
	PPMI	4.0 ± 2.8 (192)	4.2 ± 2.9 (150)	4.4 ± 3.2 (143)	4.2 ± 2.8 (97)	0.77
SCOPA-Aut Score, mean ± SD (count)	ENIGMA-PD	0.0 (1)	8.0 (1)	15.2 ± 8.3 (6)	16.7 ± 11.0 (3)	0.37
	PPMI	10.1 ± 5.1 (55)	11.6 ± 8.2 (41)	12.1 ± 5.8 (37)	8.2 ± 4.8 (12)	0.27
UPSIT score, mean ± SD (count)	ENIGMA-PD			11 (1)	34 ± 1 (1)	0.32
	PPMI	18.9 ± 11.2 (192)	19.6 ± 11.7 (152)	17.2 ± 11.4 (143)	19.6 ± 9.3 (97)	0.25

^a^Bold *P*-values indicate a *P* ≤ 0.05. Kruskal–Wallis test for all, except ‘Count’, ‘% of Females’, and ‘Hoehn and Yahr Stage’, which used Pearson’s *χ*^2^. Statistical tests are all performed across subtypes within the same dataset, apart from ‘Count’, where ENIGMA-PD and PPMI are compared.

Bold data values indicate the subtypes that were significantly different from each other in the *post hoc* Conover’s statistical test with Bonferroni correction for the number of subtypes. This was calculated for all characteristics apart from ‘Count’ and ‘Hoehn and Yahr Stage’. The values for ‘Years of Education’ in PPMI are omitted as the sample sizes are too small.

ENIGMA-PD, Enhancing Neuroimaging through Meta-Analysis consortium Parkinson’s Disease; MDS-UPDRS, Movement Disorders Society Unified Parkinson’s disease Rating Scale; MoCA, Montreal Cognitive Assessment; PPMI, Parkinson’s Progression Markers Initiative; SD, standard deviation; MAD, median absolute deviation.

In the test set (not analysed in the training set), no statistically significant differences between subgroups were observed for the number of individuals assigned tremor dominant or postural instability with gait disorder (Supplementary Table 1). No significant differences between subgroups were found in baseline clinical scores in the test data. Unlike the PPMI dataset, some small yet significant subtype differences were observed in the ENIGMA-PD training data: MoCA was 1.2 points lower in the *Limbic* subtype (*P* < 0.01), and Hoehn and Yahr Stage was slightly lower (<1 stage) in the *Sub-threshold atrophy* group (*P* < 0.001). Site (available only in the training set) was statistically associated with atrophy model subgroup (Pearsons’s χ^2^, *P* < 0.001). Supplementary Fig. 4 visualizes subtype assignment by cohort within the ENIGMA-PD dataset.

Supplementary Table 2 reports statistical comparisons between the *Sub-threshold atrophy* group (minimal/zero atrophy) against all subtypes as a single group (any atrophy). The subtyped group was at least 1 year older in both the training and test sets (*P* ≤ 0.02) and had a longer disease duration (*P* < 0.01). The subtyped group in the test data was on average >4 years older at diagnosis (*P* < 0.001) and had a higher MDS-UPDRS-III by almost 2 points on average (*P* = 0.02). The subtyped group in the training data had a slightly higher Hoehn and Yahr Stage (*P* < 0.001).

### Longitudinal subgroup analysis: PPMI

The longitudinal data in PPMI facilitates an analysis of disease progression across the atrophy subtypes and *Sub-threshold atrophy* subgroup, for which we performed linear regression and survival analyses (see Methods).

Supplementary Fig. 5 shows MoCA data and linear fits for each subgroup in PPMI for up to 12 years from baseline and separately for only 4 years from baseline to allow for attrition. We found no statistically significant differences in cognitive decline between subgroups over 12 years, despite some people with Parkinson’s having lower global cognitive scores (MoCA < 26).^77^ This is likely driven by long-term attrition of decliners, since the same analysis over only 4 years showed the Limbic subtype having a statistically significant decline in MoCA of approximately 0.4 points per year. While we observed clinical disease progression in other measures, e.g. MDS-UPDRS-III (‘off state’) motor scores (Supplementary Fig. 6), there were no clinically meaningful differences between subgroups, including no differences in more detailed cognitive tests nor MoCA sub-scores.

Supplementary Fig. 7 shows reasons for withdrawal from the PPMI study (test set). Subgroup-specific counts of withdrawal reasons showed no statistically significant subgroup bias.

**Figure 6:**
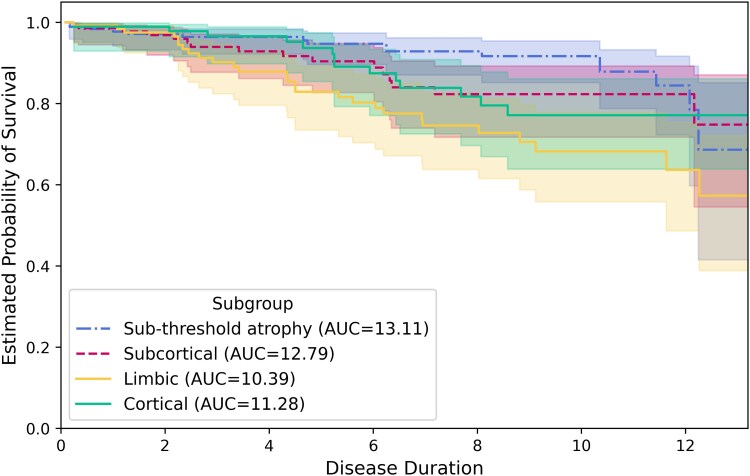
**Survival analysis for cognitive decline or study withdrawal (PPMI test data).** Kaplan–Meier curves for events including MoCA *<* 21 or withdrawal for any of the following reasons: burden of study procedures (other than travel), decline in health, institutionalized and death. MoCA < 21 has previously been found to be a suitable cutoff for Parkinson’s disease dementia detection.^77,78^ At a disease duration of 0 years, *N* = 172, 131, 108 and 83, for the *Sub-threshold atrophy* (blue dot-dash line)*, Subcortical* (red dashed line)*, Limbic* (yellow solid line) and *Cortical* (green solid line) subgroups, respectively. At a disease duration of 13 years, *N* = 156, 115, 81 and 69, for the *Sub-threshold atrophy, Subcortical, Limbic* and *Cortical* subgroups, respectively. MoCA, Montreal Cognitive Assessment; AUC, area under the curve.

Figure 6 is a plot of Kaplan–Meier curves showing that the *Limbic* subgroup exhibited visibly poorer survival than the others, with the *Sub-threshold atrophy* subgroup (*P* < 0.005 against the *Limbic* group, *P* ≥ 0.05 for all other comparisons) having the best survival outcomes when not adjusting for covariates (log-rank test, Supplementary Table 3). However, when adjusting for covariates, as seen in the Cox Proportional Hazards model results in Supplementary Table 4, the results obtained are slightly different. The *Cortical* subgroup had the poorest survival compared with all other subgroups combined. Similarly, the *Limbic* subgroup also showed a trend towards statistically different survival curves to the *Sub-threshold atrophy* subgroup when adjusting for covariates (*P* = 0.06, Cox Proportional Hazards). When comparing the *Sub-threshold atrophy* subgroup to all subtypes (combined), a statistical difference was found when not adjusting for covariates (*P* < 0.005, log-rank test), but not when comparing any of the other subgroups or adjusting for covariates (*P* = 0.20, Cox Proportional Hazards).

## Discussion

In this study, we applied SuStaIn—a robust, state of the art disease progression clustering algorithm—to discover T1w-MRI atrophy subtypes in the world’s largest Parkinson’s disease dataset, collated by the ENIGMA-PD working group. We tested the model on separate, longitudinal data from the PPMI study. Despite some evidence that cortical thinning and subcortical volumetric changes are relatively late events in Parkinson’s disease progression,^80^ three longitudinally-robust subtypes were discovered and validated, characterised by distinct spatiotemporal patterns of group-level atrophy: early *Subcortical* atrophy; early *Cortical* thinning; and *Limbic*-predominant atrophy. A fourth subgroup, called *Sub-threshold atrophy*, showed sub-threshold atrophy across the entire brain (below a normative *z*-score of 0.5).

Our *post hoc* analysis of the three atrophy subtypes produced mixed results, with only a few statistically significant differences found. For instance, we found poorer survival in the *Limbic* subtype versus the *Sub-threshold atrophy* subtype. Further work is needed to investigate whether the atrophy subtypes discovered by SuStaIn reflect underlying and distinct pathogenic mechanisms.^81^ Clinically, one might hypothesize subtype–phenotype relationships based on the group-level atrophy patterns discovered, e.g. a cognition-dominated clinical phenotype in the limbic-predominant atrophy subtype relative to others. We found only subtle cognitive differences between subgroups using a combined cognitive/adverse-event survival analysis. In the PPMI test set, the *Sub-threshold atrophy* group had subtly superior survival of cognition (using a composite MoCA and dropout outcome). Regression analysis suggests that only the *Limbic* subtype displays statistically significant cognitive decline. These results overall suggest a possible survival benefit of having minimal atrophy. Future work involving longer follow-up is needed to validate and fully assess the longitudinal implications of this finding.

A possible explanation for the absence of strong subtype/clinical-phenotype associations for our image-based subtypes, despite the widely documented clinical heterogeneity of Parkinson’s disease, is that macroscopic brain atrophy measured by T1w MRI may be insensitive to the biological underpinnings of the clinical phenotype. Indeed, previous work suggests the superiority of other, usually quantitative, brain imaging modalities being more sensitive to Parkinson’s disease-related changes than T1w MRI, e.g. quantitative susceptibility mapping^17,82-84^ and diffusion MRI.^85-88^ Such non-T1w neuroimaging data are only sparsely available in subsets of the ENIGMA-PD dataset used here for model training (image-based subtype discovery) and in clinical practice. In contrast, T1w MRI scans are routinely performed, warranting this investigation as a first step.

Additionally, our longitudinal results may be confounded by the unique features of the PPMI dataset. PPMI is a research cohort with recruitment early in the disease course and may represent people with Parkinson’s who have milder disease and less severe disease progression. For instance, it has already been observed that PPMI participants have a lower rate of dementia diagnosis compared with other research studies.^89^ Our own finding of PPMI having a greater proportion of *Sub-threshold atrophy* participants supports this. This unique characteristic of the PPMI dataset may be a contributing factor to the limited cognitive differences we observed between subgroups longitudinally.

The subtypes we found (*Limbic*, *Cortical* and *Subcortical*) are qualitatively similar to those produced in SuStaIn analyses of other diseases.^14,90^ One might ask whether these subtypes reflect natural variation rather than neurodegenerative disease. Alternatively, this might reflect some fundamental biological factor shared among neurodegenerative diseases, such as selective vulnerability.^91^ Detailed investigation of this is beyond the scope of this paper.

Most previous Parkinson’s disease subtyping studies were performed in ‘reverse’ compared with our study: non-clinical (e.g. neuroimaging) associations between clinical-based subtypes.^30-35,40,44-46,92^ Our motivation was the reverse: to explore the possibility of *post hoc* clinical associations in image-based subtypes. The ultimate clinical application for such imaging subtypes would be deployment for pre-symptomatic clinical decision support, e.g. stratification for clinical trials and prognosis. This remains an important goal, but our study suggests that subtype and stage inference performed on measures of subcortical brain volumes and cortical thicknesses from T1w MRI may not be adequate for this purpose.

Previous studies on image-based subtype discovery have involved much smaller datasets and have primarily found cortical atrophy signatures.^93^ Some previous studies found significant associations between image-based subtypes and clinical phenotypes, and we discuss examples in the following paragraphs.

Uribe *et al.*  ^94^ (*n* = 88) found three atrophy patterns: parieto-temporal having worse cognitive performance, occipital and frontal having younger disease onset and individuals without detectable cortical atrophy. The latter group agrees with our findings of a substantial *Sub-threshold atrophy* population and the *Cortical* Subtype we found starts with parieto-temporal atrophy. The atrophy pattern of the *Cortical* Subtype also matches that observed more generally in people with Parkinson’s disease dementia^95,96^ and people with Alzheimer’s disease.^97^ Despite this, our *Cortical* Subtype showed no significant association with cognitive decline, probably due to attrition when severe motor symptoms eventually preclude study participation.

Other notable atrophy-based subtyping studies found clinical associations in image-based subtypes, although in smaller sample sizes than those in our study. Inguanzo *et al.*^98^ (*n* = 633) found eight brain patterns of atrophy when adjusting for global atrophy, although these were not found consistently in individual cohorts. When not adjusting for global atrophy, three subtypes were identified, with two having high levels of atrophy and resembling different Braak stages: no detectable atrophy, atrophy in the amygdala and neocortical atrophy. Pan *et al.*^99^ (*n* = 107) found two subtypes based on brain atrophy rates, with the faster atrophy subtype (particularly in the lateral temporal region) showing worse clinical decline. It remains uncertain whether these findings are coincidental, possibly resulting from the relatively small sample sizes (ranging from 88 to 633 for the cited works, compared with 1100 in our study).

Recently, Sakato *et al*.^100^ similarly found three subtypes of progression using SuStaIn on three cohorts (*n* = 504, with an overall older cohort, with longer disease duration but also including PPMI): a neocortical, limbic and brainstem. The pattern of neurodegeneration in the brainstem subtype resembles that of the subcortical subtype found in our analysis. In contrast to our research, they used larger *z*-scores (*z* = 12,3) as stage progression criteria and only analysed clinical outcomes at baseline (rather than longitudinally). The subtypes they found had significantly different age and age of onset at baseline. However, all other phenotypic statistical differences were against individuals who were not assigned a subtype (stage 0). Therefore, the findings from both our study and theirs support the notion that atrophy severity is more predictive of poor prognosis in Parkinson's disease than atrophy location.

### Limitations and future work

There are several potential explanations for why our study found no strong subtype/clinical-phenotype associations. Beyond the survivor bias inherent to longitudinal studies of neurodegenerative diseases, the most plausible reason is that macroscopic atrophy detectable on T1w MRI may be inadequate for unravelling the biological heterogeneity of Parkinson’s disease for clinical use up to 12 years post-diagnosis (subject to survivor bias). Although not available in our datasets, sensible avenues of future data-driven subtype discovery work include using multimodal biomarkers (e.g. quantitative imaging), fluid biomarkers, genetic markers and advanced clinical assessments like vision-tests, presence of REM sleep behaviour disorder, dream content, etc.—all of which have been shown to contain disease signal in Parkinson’s disease.^17,88,101-104^ In particular, co-pathologies such as beta-amyloid and tau accumulations are likely to contribute to rates of cognitive decline in Parkinson’s disease.^105^ Future work that includes biomarkers sensitive to these co-pathologies may reveal subtypes of progression that differ depending on relative levels of these co-pathologies.

Clustering can be confounded by non-biological variance, i.e. noise. Relevant to T1w MRI, this includes FreeSurfer variability,^106-108^ imaging quality/artifacts, and batch effects.^109^ Indeed, our multi-cohort training dataset assembled via the ENIGMA-PD working group includes many sub-cohorts, which are often multi-site studies themselves. Although there were imaging protocol similarities, there was no standardization of data collection. However, each data contributor was requested to use the same major version of FreeSurfer (v5) to process the MRI, which can help to reduce the known variability in estimated cortical thickness and subcortical volume values.^106-108^ Future work could include data harmonization to remove batch effects,^65,109,110^ or the logistically more challenging data centralization and singular processing to reduce the possible confounding influence of non-biological variability. We also observed a high level of missingness in the ENIGMA-PD data (Supplementary Fig. 8), but the reasons behind this are unknown. Future work might investigate this further to ensure no disease-relevant selection bias has been introduced.

Dropout (or attrition) over the 12 years of data in the PPMI test set will impede our ability to detect longitudinal subtype/clinical-phenotype associations. Since dropout can be due to clinical decline, we attempted to investigate this statistically using a survival analysis that included cognitive decline and key reasons for dropout among the events. This showed subtle (trend-level) differences among subtypes when adjusting for covariates, with the Limbic and Subcortical subtypes faring the worst compared with having Sub-threshold atrophy. These differences may have been amplified by including broader neuropsychiatric symptoms, such as moderate to severe hallucinations or apathy, as seen in work by Brumm *et al*.^111^ Alternatively, future work to remedy this might require very long prospective studies with a focus on minimising attrition—possibly unattainable in a research setting, but possibly feasible via a population-based study in a healthcare setting, since additional longitudinal outcomes (even if not detailed clinical testing) are more likely to be available.

The datasets used here may not wholly reflect clinical populations of people with Parkinson’s disease; ENIGMA has both clinical populations and research cohorts, while PPMI is a research cohort with milder disease progression.^89^ A future population-based study could investigate whether the subtypes found here are consistent with clinical populations.

There are documented differences in longitudinal outcomes between early-onset and late-onset Parkinson’s disease, despite no consensus on the best cutoff age at onset. Our analysis regressed out age-related effects, but did not attempt to account for age at onset. Age at onset did not vary considerably between our image-based subtypes, which supports this experimental design choice and the notion that age-at-onset differences in (T1w MRI-based) atrophy profile are negligible. However, there might be value in training separate later-onset and earlier-onset Parkinson’s disease subtype models—particularly on multimodal data since later-onset individuals have an elevated risk of dementia, and multimodal non-subtype models have shown promise.^17^

### Conclusion

We have reported a data-driven computational model of three atrophy subtypes (and one sub-threshold atrophy subgroup) of Parkinson’s disease trained on a uniquely large and diverse multi-cohort dataset. The model was longitudinally validated and tested on a separate large, multi-site dataset. Clinical phenotypes (cognitive, motor, etc.) of the three atrophy subtypes were, at best, subtly different over up to 12 years, suggesting that the macroscopic atrophy measured by T1w MRI imaging may not provide a useful (pre)clinical stratification for people with Parkinson’s disease. Clinical statistical differences were found between the *Sub-threshold atrophy* group and the atrophy subtypes. This indicates that atrophy severity rather than atrophy location is a more informative differentiator of Parkinson’s disease. As new biomarkers and techniques are emerging, such as α-synuclein seeding amplification assays,^8,112,113^ PET-tracers,^114^ and tissue-sensitive MRI approaches such as quantitative susceptibility mapping,^82,83,115^ we are optimistic that data-driven subtyping methods can eventually be combined with the right data to contribute useful insights in Parkinson’s disease prognosis and for aiding the search for disease modifying treatments.^116^

## Supplementary Material

fcaf146_Supplementary_Data

## Data Availability

Individual ENIGMA-PD sites retain ownership of their MRI scans and only shared the anonymized derived data for analysis. Data are thus not openly available, but researchers are invited to join the ENIGMA-PD Working Group where they can formally request derived data via secondary proposals. Data requests are then considered by the individual site's principal investigators. If you are interested in joining ENIGMA-PD, please contact enigma-pd@amsterdamumc.nl. For more information please see the working group website: https://enigma.ini.usc.edu/ongoing/enigma-parkinsons/. Data used in the preparation of this article were obtained on 2024-01-01 (quarterly data freeze) from the Parkinson’s Progression Markers Initiative (PPMI) database (www.ppmi-info.org/access-data-specimens/downloaddata), RRID:SCR 006431. For up-to-date information on the study, visit www.ppmi-info.org. The Python implementation of SuStaIn is publicly available at: https://github.com/ucl-pond/pySuStaIn. pySuStaIn version 0.1 was used for this research.

## Appendix

ENIGMA-PD working group members:

Max A. Laansma, Joanna K. Bright, Sarah Al-Bachari, Tim J. Anderson, Tyler Ard, Francesca Assogna, Katherine A. Baquero, Henk W. Berendse, Benajmin Newman, Fernando Cendes, John C. Dalrymple-Alford, Rob M. A. de Bie, Ines Debove, Michiel F. Dirkx, Jason Druzgal, Hedley C. A. Emsley, Gäetan Garraux, Rachel P. Guimarães, Boris A. Gutman, Rick C. Helmich, Johannes C. Klein, Clare E. Mackay, Corey T. McMillan, Tracy R. Melzer, Laura M. Parkes, Fabrizio Piras, Toni L. Pitcher, Kathleen L. Poston, Mario Rango, Letícia F. Ribeiro, Cristiane S. Rocha, Christian Rummel, Lucas S. R. Santos, Reinhold Schmidt, Petra Schwingenschuh, Gianfranco Spalletta, Letizia Squarcina, Odile A. van den Heuvel, Chris Vriend, Jiun-Jie Wang, Daniel Weintraub, Roland Wiest, Clarissa L. Yasuda, Neda Jahanshad, Paul M. Thompson, Ysbrand D. van der Werf.
