# Supplementary material for: Neuroimaging-based data-driven subtypes of spatiotemporal atrophy due to Parkinson’s disease
Source: Brain Commun. 2025 Apr 16;7(2):fcaf146. doi: 10.1093/braincomms/fcaf146 (PMC12037470; doi:10.1093/braincomms/fcaf146)
Supplement: fcaf146_Supplementary_Data [file fcaf146_supplementary_data.docx]

# Supplementary Material

## Linear Mixed Regression Models

The linear mixed regression models^1^ were created using the following formula format in the Python *statsmodels* package:

*clinical feature* ∼ −1 + *ml_subtype*(*Years_bl* + 1) (2)

where *clinical feature* is a given feature being tested, such as MoCA, *ml_subtype* is the subtype assignment with the highest likelihood for a given patient and *Years_bl* is the number of years since baseline.

## CVIC and Out-of-sample Log Likelihoods


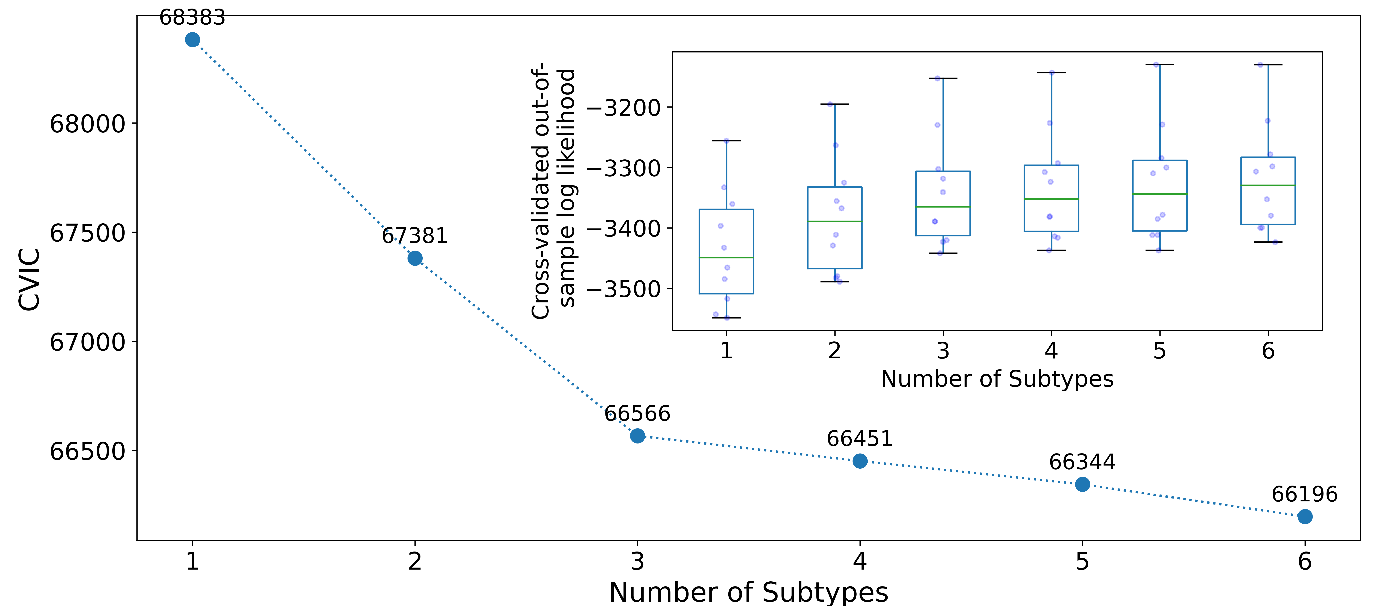


**Supplementary Figure 1:** **Cross-validation results during SuStaIn training.** CVIC shows diminishing returns for *N >* 3 subtypes, as does out-of-sample log-likelihood (inset). Each data point in the out-of-sample log-likelihood, represents the results from one cross-validation fold (therefore as we performed 10-fold cross-validation, each box has 10 data points). *Abbreviations —* SuStaIn: Subtype and Stage Inference; CVIC: Cross-validation information criterion.


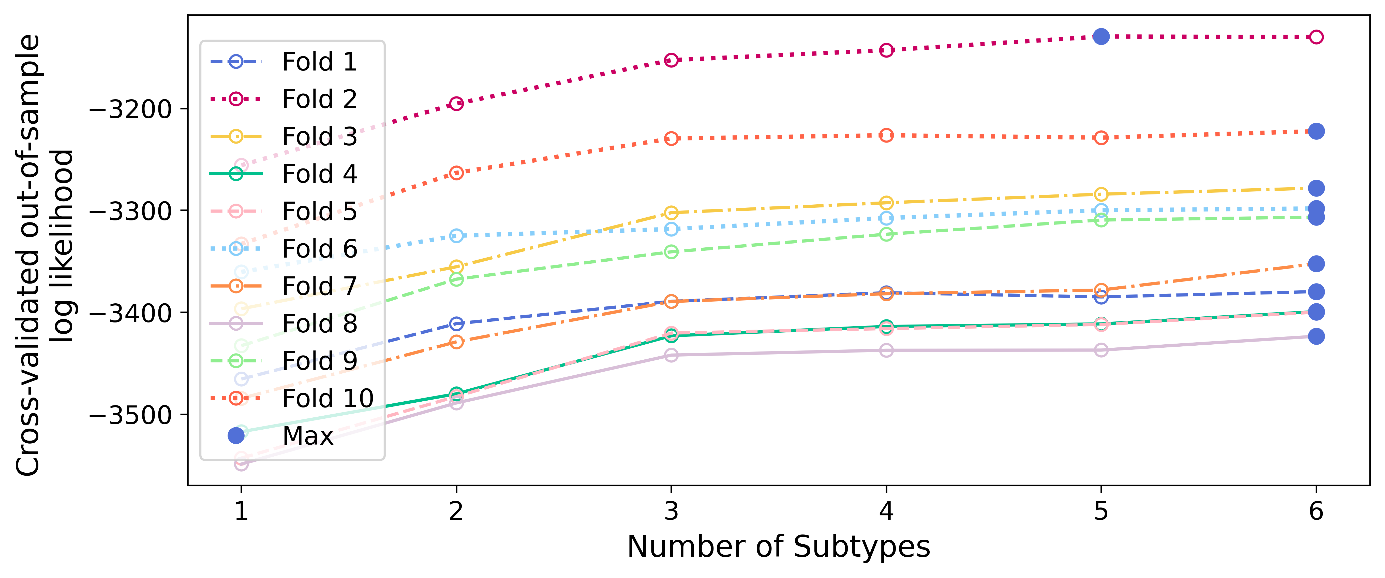


**Supplementary Figure 2: Cross-validated out-of-sample log likelihoods for the different number of potential subtypes for ENIGMA-PD.** The maximum log likelihood differs depending on the fold and more folds agree on a 9-subtype model fitting the data best. The log likelihoods plateau at the 3-subtype model for all folds. There are 10 lines in total, one for each cross-validation fold within each N-subtypes model (x-axis). For each model (from N=1 to 6 subtypes), the cross-validated out-of-sample log-likelihood is shown for each of the 10 folds. Therefore, each line has 6 data points. *Abbreviations —* PD: Parkinson’s disease; ENIGMA: Enhancing Neuroimaging through Meta-Analysis.

## Other Results

**Supplementary Table 1: TD/PIGD Classification of PPMI individuals in each subgroup**

| **Subgroup** | **Indeterminate (%)** | **PIGD (%)** | **TD (%)** |
| --- | --- | --- | --- |
| ***Sub-threshold atrophy*** | 23 (12) | 41 (21) | 128 (67) |
| ***Subcortical*** | 30 (20) | 25 (16) | 97 (64) |
| ***Limbic*** | 18 (13) | 27 (19) | 98 (69) |
| ***Cortical*** | 13 (13) | 25 (26) | 59 (61) |

*Abbreviations —* TD: Tremor Dominant; PIGD: Postural instability and gait difficulty; PPMI: Parkinson’s Progression Markers Initiative; SD: standard deviation.

**Supplementary Table 2: Clinical Features for *Sub-threshold atrophy* and Subtyped Patients at Baseline**

| **Characteristic** | **Dataset** | ***Sub-threshold atrophy*** | **Subtyped** | **P-value*** |
| --- | --- | --- | --- | --- |
| Count | ENIGMA-PD | 317  192 | 783  392 | *<* **0.001**  *<* **0.001** |
|  | PPMI |  |  |  |
| % Female | ENIGMA-PD  PPMI | 38.8  34.9 | 37.2  41.6 | 0.66  0.14 |
| Age,  mean ± SD (count) | ENIGMA-PD | 62.2 ± 8.6 (317)  60.2 ± 9.8 (192) | 63.5 ± 9.5 (783) | **0.02** |
|  | PPMI |  | 64.5 ± 9.1 (392) | *<* **0.001** |
| Age of Onset,  mean ± SD (count) | ENIGMA-PD  PPMI | 57.0 ± 9.9 (207)  59.3 ± 9.9 (189) | 56.3 ± 11.3 (510)  63.4 ± 9.1 (382) | 0.76  *<* **0.001** |
| Disease Duration,  years ± SD (count) | ENIGMA-PD  PPMI | 6.4 ± 4.9 (249)  0.8 ± 1.1 (189) | 7.8 ± 5.6 (622)  1.0 ± 1.2 (382) | **< 0.001**  **< 0.01** |
| Hoehn and Yahr Stage,  median [MAD] (count) | ENIGMA-PD  PPMI | 2.0 [0.0] (199)  2.0 [0.0] (190) | 2.0 [0.0] (531)  2.0 [0.0] (390) | *<* **0.001**  0.24 |
| MoCA Score,  mean ± SD (count) | ENIGMA-PD  PPMI | 24.3 ± 4.4 (177)  27.2 ± 2.5 (190) | 23.3 ± 5.1(411)  26.8 ± 2.6 (383) | 0.98  0.96 |
| MDS-UPDRS-III,  mean ± SD (count) | ENIGMA-PD  PPMI | 29.9 ± 13.8 (43)  20.9 ± 9.3 (189) | 32.6 ± 15.2 (75)  22.8 ± 10.2 (390) | 0.17  **0.02** |
| RBD Score,  mean ± SD (count) | ENIGMA-PD  PPMI | -  4.0 ± 2.8 (192) | -  4.3 ± 3.0 (390) | **-**  0.15 |
| SCOPA-Aut Score,  mean ± SD (count) | ENIGMA-PD  PPMI | 0.0 (1)  10.1 ± 5.1 (55) | 14.9 ± 8.5 (10)  11.3 ± 6.9 (90) | 0.09  0.31 |
| UPSIT Score,  mean ± SD (count) | ENIGMA-PD  PPMI | -  18.9 ± 11.2 (192) | 22.5 ± 16.3 (2)  18.7 ± 11.1 (392) | -  0.57 |

- **Bolded text** indicates a p-value ≤ 0.05. Mann-Whitney U test for all, apart from “Count”, “% Female”, and “Hoehn and Yahr Stage” which was obtained with a Pearson’s *χ*^2^.

*Abbreviations —* ENIGMA-PD: Enhancing Neuroimaging through Meta-Analysis consortium Parkinson’s Disease; MDS-UPDRS: Movement Disorders Society Unified Parkinson’s disease Rating Scale; MoCA: Montreal Cognitive Assessment; PPMI: Parkinson’s Progression Markers Initiative; SD: standard deviation; MAD: median absolute deviation.


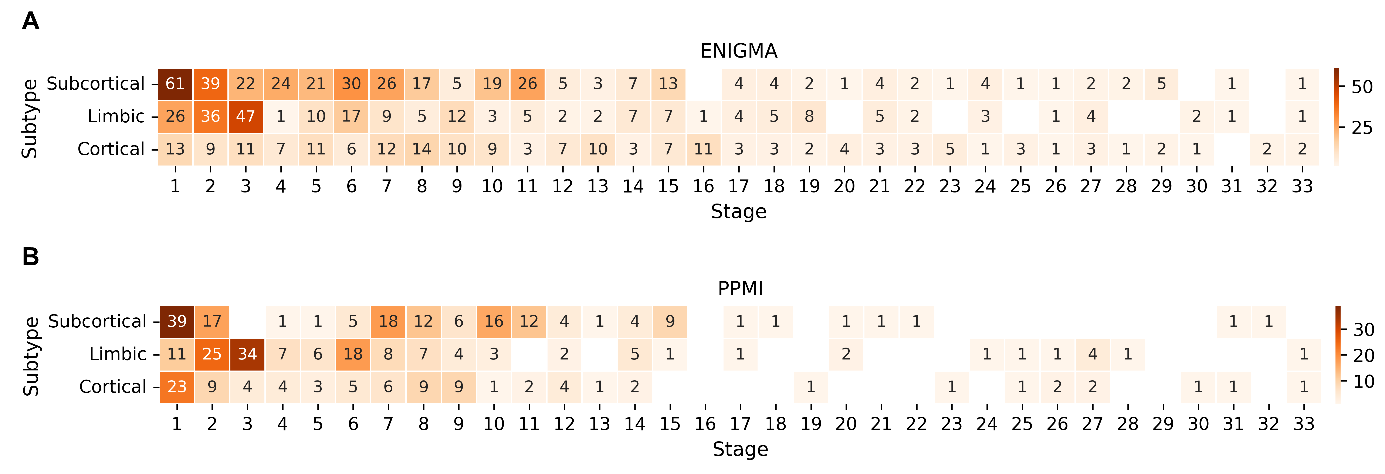


**Supplementary Figure 3: Heatmaps showing the number of people with Parkinson’s disease per subtype (y-axis) and stage (x-axis) in (A) ENIGMA-PD and (B) PPMI.** The number of individuals in each cell is indicated by the number on and colour of that cell. If there are no individuals populating a given stage and subtype, the cell remains empty. In all subtypes, most people with Parkinson’s disease are staged early. The Subcortical subtype has the largest number of participants in both ENIGMA (N = 359) and PPMI (N = 152). The Limbic subtype is the second most populated subtype in ENIGMA (N = 237) and in PPMI (N = 143) as well. Lastly, the Cortical subtype had the smallest number of participants with N = 187 in ENIGMA and N = 97 in PPMI. *Abbreviations —*ENIGMA: Enhancing Neuroimaging through Meta-Analysis; PPMI: Parkinson’s Progression Markers Initiative.


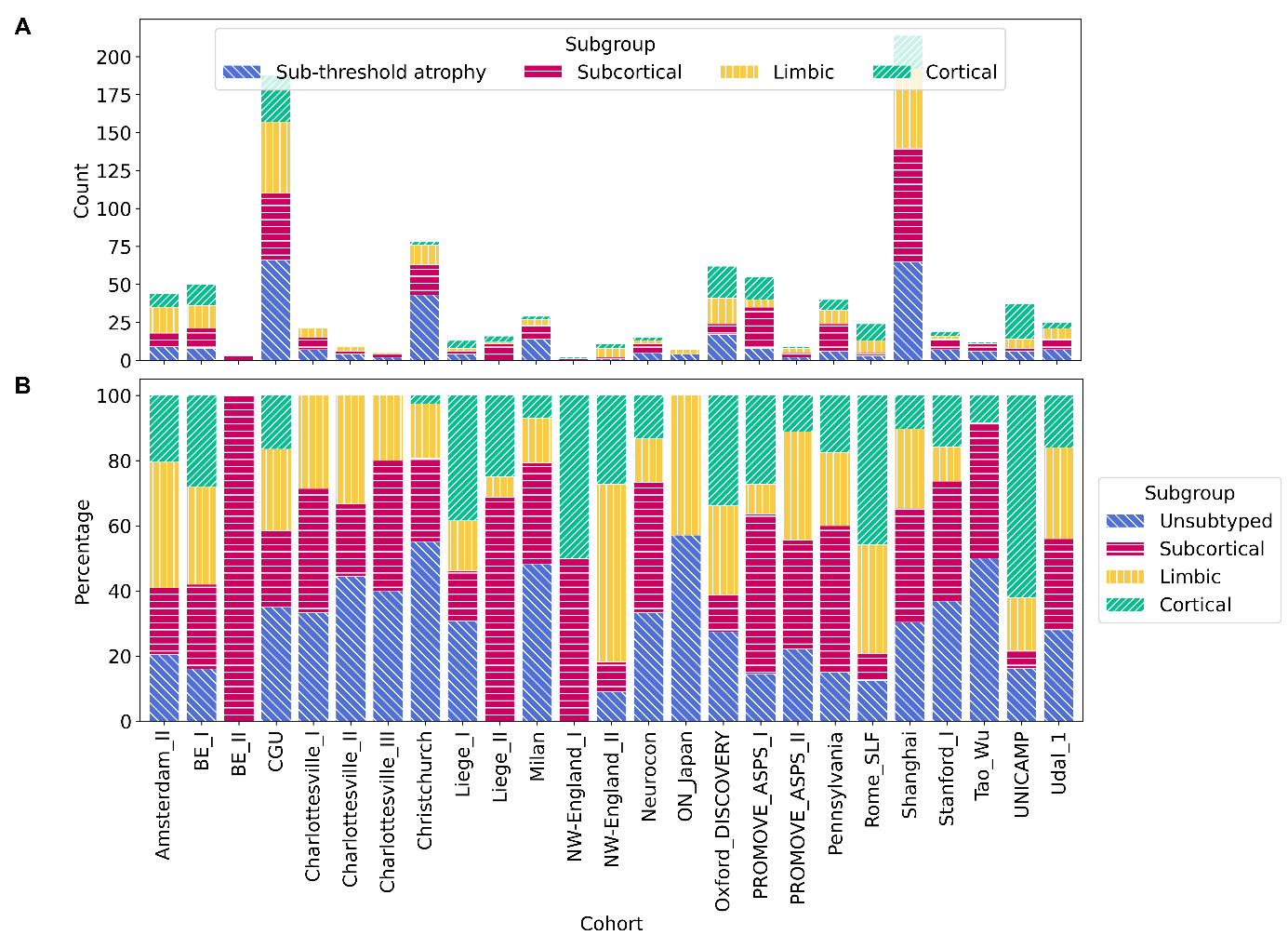


**Supplementary Figure 4: (A)** A stacked bar plot highlighting the number of individuals per cohort in ENIGMA-PD and their model subgroup assignment (total N = 998). The subgroups are highlighted by the different fill patterns shown in the legend. (**B)** Is a stacked bar plot showing the percentage of individuals assigned to a certain subgroup per cohort. The majority of participants are from two cohorts: CGU and Shanghai. The variation in subtype proportions between cohorts may reflect demographic differences in this diverse international dataset. *Abbreviations —* PD: Parkinson’s disease; ENIGMA: Enhancing Neuroimaging through Meta-Analysis; BE: Bern; CGU: Chang Gung University; NW: North-West; PROMOVE_ASPS: Prospective Movement Disorders Registry, Austrian Stroke Prevention Study, Graz; SLF: Santa Lucia Foundation; UNICAMP: State University of Campinas.


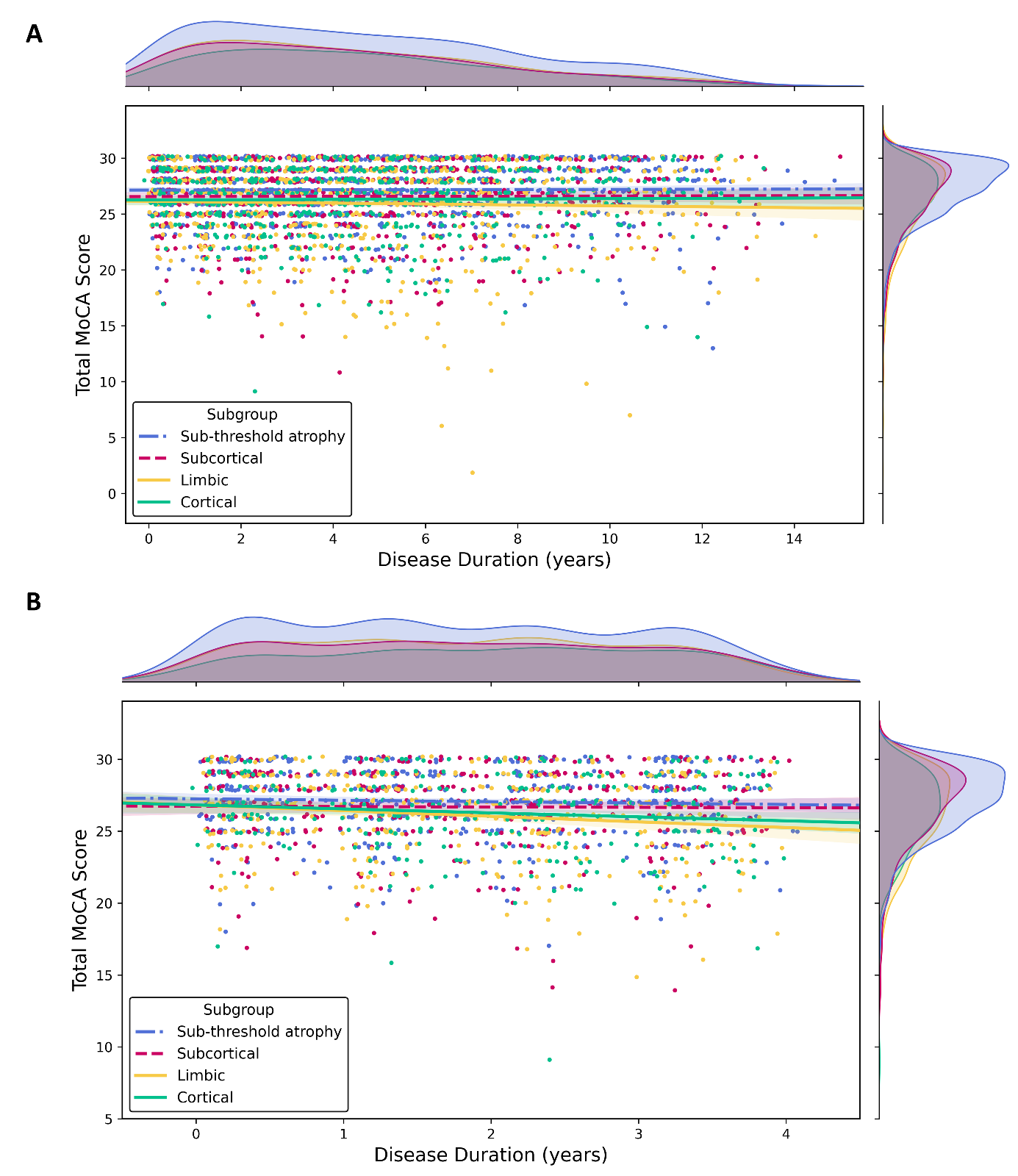


**Supplementary Figure 5:** **Longitudinal MoCA scores in Parkinson’s disease subtypes (PPMI test data) (A) For all longitudinal data. (B) Up to a disease duration of 4 years.** Each plotted point is a participant at a specific time in their disease course (N = 3233 data points from 568 participants for (A) and N = 1573 data points from 545 participants for (B)). Participants have been assigned a subtype based on their baseline neuroimaging data. Linear regression showed minimal cognitive change over 9 years in all subgroups (atrophy subtypes plus *Sub-threshold atrophy*). Greater differences are observed when only examining participants with a disease duration of 4 years maximum, with the Limbic (gradient and confidence interval of -0.39 ± 0.27, p<0.005) and Cortical subtypes (gradient and confidence interval of -0.28 ± 0.64, p=0.055) showing statistically significant gradients. This is potentially because of dropout bias, as participants with greater cognitive decline leave the study earlier. Therefore, any modelling, significantly past 5 years would most likely include participants with cognitive resilience, rather than decline. *Abbreviations —* MoCA: Montreal Cognitive Assessment; PPMI: Parkinson’s Progression Markers Initiative.


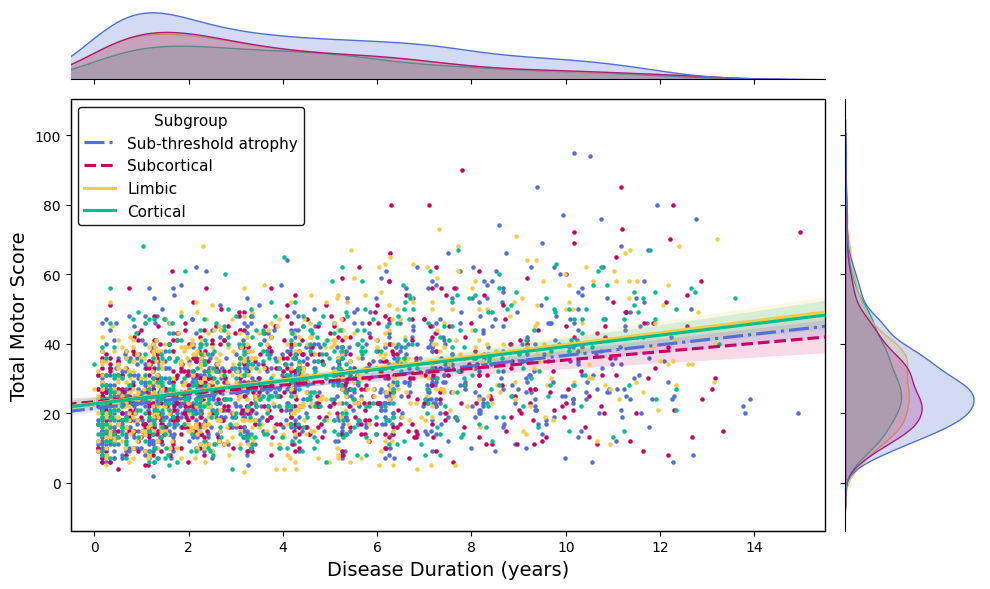


**Supplementary Figure 6: A line and scatter plot of the total motor scores, or MDS-UPDRS-III scores, over time per subgroup.** Each plotted point is a participant at a specific time in their disease course (N=3001 data points from 569 participants). Participants have been assigned a subtype based on their baseline neuroimaging data. The lines show the gradient, or change over time, in MDS-UPDRS-III scores per subgroup, with no statistically significant differences found (p>0.05, in a mixed linear model regression z-test for mixed effects). The shaded areas show the corresponding uncertainty associated with the calculated gradient. *Abbreviations —*PPMI: Parkinson’s Progression Markers Initiative.

**
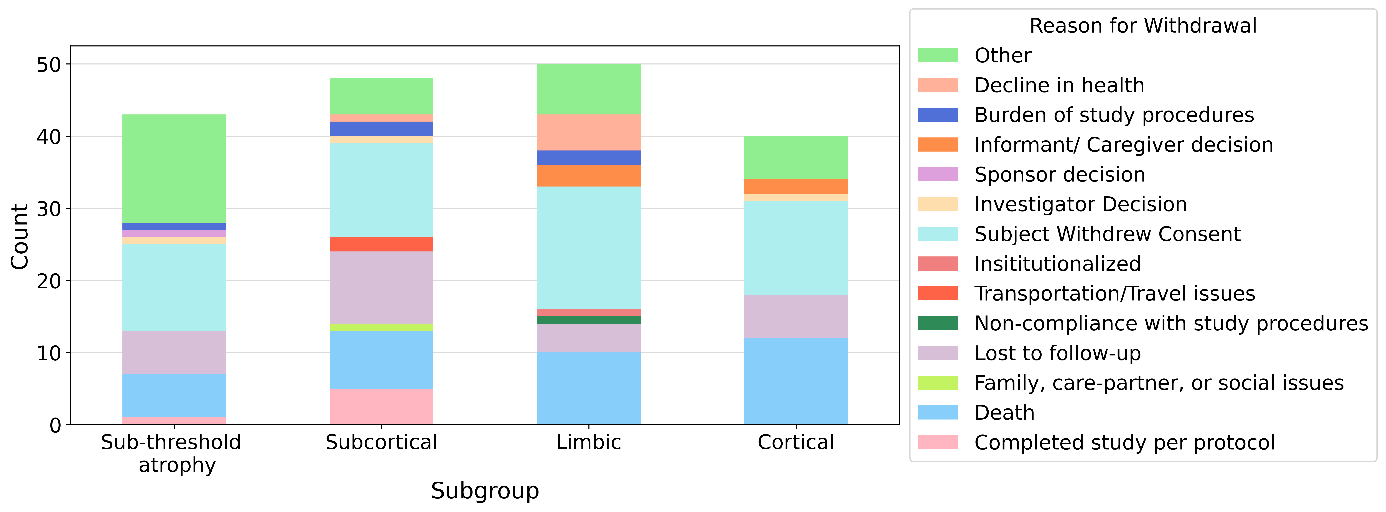
**

**Supplementary Figure 7:** **Reasons for study withdrawal by data-driven subgroup (PPMI test data.** A stacked bar chart showing the reasons for withdrawal per subgroup in the PPMI cohort (N = 181). *Abbreviations —*PPMI: Parkinson’s Progression Markers Initiative.

### Survival Analysis Statistical Testing

#### Log-Rank Test

**Supplementary Table 3: Comparison of Subgroups Survival via a Log-Rank Test**

| **Comparison** | **Test Statistic** | **P-value^1^** |
| --- | --- | --- |
| ***Sub-threshold***  ***atrophy* vs. *All*** | 10.21 | **<0.005** |
| ***Sub-threshold atrophy* vs. *Subcortical*** | 1.20 | 0.27 |
| ***Sub-threshold atrophy* vs. *Limbic*** | 10.21 | **<0.005** |
| ***Sub-threshold atrophy* vs. *Cortical*** | 2.43 | 0.12 |
| ***Subcortical* vs. *Limbic*** | 3.30 | 0.07 |
| ***Subcortical* vs. *Cortical*** | 0.01 | 0.91 |
| ***Limbic* vs. *Cortical*** | 2.60 | 0.11 |

**^1^Bolded text** indicates a p-value ≤ 0.05.

#### Cox Proportional Hazards Model

**Supplementary Table 4: Cox Proportional Hazards Model Results**

| **Subgroups compared** | **Model Coefficients (p-value)^1^** | | | ***Concordance*** | ***Partial AIC*** | **Log-likelihood ratio test** |
| --- | --- | --- | --- | --- | --- | --- |
|  | **Sex** | ***Subtype*** | ***Stratified Age of Onset*** |  |  |  |
| **Sub-threshold atrophy vs. All** | 0.04 (0.86) | *0.10 (0.20)* | ***-1.29 (<0.005)*** | 0.67 | 791.81 | 32.63 |
| **Sub-threshold atrophy vs. Subcortical** | 0.48 (0.20) | *0.29 (0.11)* | ***-1.55 (<0.005)*** | 0.71 | 307.09 | 20.72 |
| **Sub-threshold atrophy vs. Limbic** | 0.04 (0.90) | *0.21 (0.06)* | ***-1.34 (<0.005)*** | 0.70 | 409.84 | 25.48 |
| **Sub-threshold atrophy vs. Cortical** | 0.34 (0.44) | *0.05 (0.62)* | ***-1.79 (<0.005)*** | 0.73 | 276.91 | 23.16 |
| **Subcortical vs. Limbic** | -0.03 (0.93) | *0.32 (0.36)* | ***-0.86 (0.01)*** | 0.62 | 416.38 | 10.12 |
| **Subcortical vs. Cortical** | 0.25 (0.54) | *-0.15 (0.47)* | ***-1.12 (<0.005)*** | 0.65 | 287.49 | 8.99 |
| **Limbic vs. Cortical** | -0.25 (0.46) | *-0.43 (0.20)* | ***-0.99 (0.01)*** | 0.65 | 384.49 | 11.95 |

**^1^ Model Coefficients and their p-values are in bold to indicate statistical significance at the p<0.05 threshold level.**

*Abbreviations —* AIC: Akaike information criterion.

## Missing Values


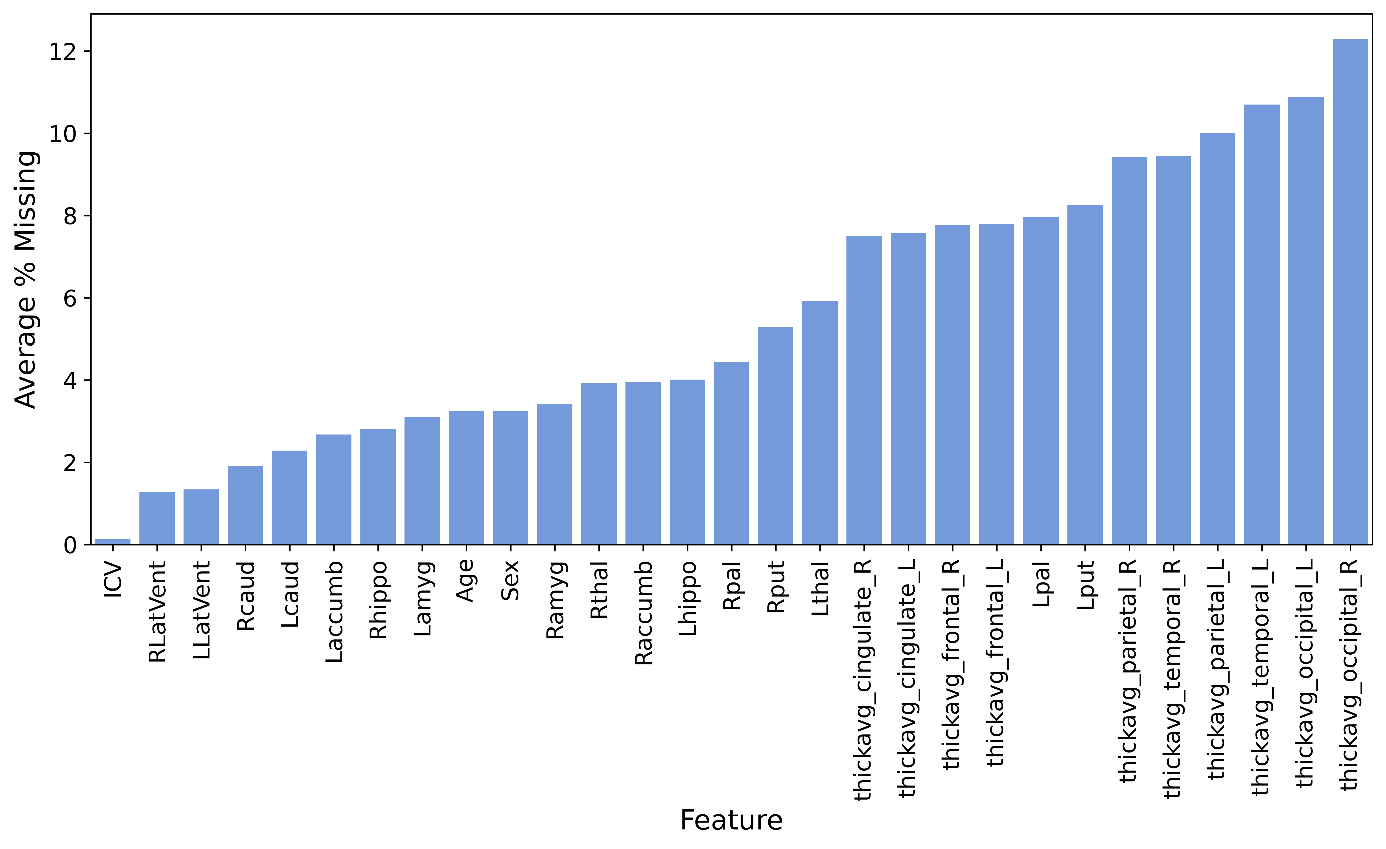


**Supplementary Figure 8: Average percentage of missing values per feature in ENIGMA-PD.** Of the N = 3009 participants with PD in ENIGMA-PD, N = 1909 had missing data in one of these features and were excluded from the main analysis. The most frequent missing feature are cortical thicknesses. *Abbreviations —* PD: Parkinson’s disease; ENIGMA: Enhancing Neuroimaging through Meta-Analysis.

# References

1. Virtanen P, Gommers R, Oliphant TE, et al. SciPy 1.0: Fundamental Algorithms for Scientific Computing in Python. *Nat Methods*. 2020;17:261-272. doi:10.1038/s41592-019-0686-2
